# Matrix-free human pluripotent stem cell manufacturing by seed train approach and intermediate cryopreservation

**DOI:** 10.1186/s13287-024-03699-z

**Published:** 2024-03-25

**Authors:** Kevin Ullmann, Felix Manstein, Wiebke Triebert, Nils Kriedemann, Annika Franke, Jana Teske, Mira Mertens, Victoria Lupanow, Gudrun Göhring, Alexandra Haase, Ulrich Martin, Robert Zweigerdt

**Affiliations:** 1 Leibniz Research Laboratories for Biotechnology and Artificial Organs (LEBAO), Department of Cardiothoracic Transplantation and Vascular Surgery (HTTG), Hannover Medical School (MHH), Carl-Neuberg-Str. 1, 30625 Hannover, Germany; 2 REBIRTH Research Center for Translational and Regenerative Medicine, Hannover Medical School (MHH), Carl-Neuberg-Str. 1, 30625 Hannover, Germany; 3 Department of Human Genetics, Hannover Medical School (MHH), Carl-Neuberg-Str. 1, 30625 Hannover, Germany

**Keywords:** hPSC, STBR, Suspension culture, Intermediate cryopreservation, Seed train, Aggregate dissociation, GMP

## Abstract

**Background:**

Human pluripotent stem cells (hPSCs) have an enormous therapeutic potential, but large quantities of cells will need to be supplied by reliable, economically viable production processes. The suspension culture (three-dimensional; 3D) of hPSCs in stirred tank bioreactors (STBRs) has enormous potential for fuelling these cell demands. In this study, the efficient long-term matrix-free suspension culture of hPSC aggregates is shown.

**Methods and results:**

STBR-controlled, chemical aggregate dissociation and optimized passage duration of 3 or 4 days promotes exponential hPSC proliferation, process efficiency and upscaling by a seed train approach. Intermediate high-density cryopreservation of suspension-derived hPSCs followed by direct STBR inoculation enabled complete omission of matrix-dependent 2D (two-dimensional) culture. Optimized 3D cultivation over 8 passages (32 days) cumulatively yielded ≈4.7 × 10^15^ cells, while maintaining hPSCs’ pluripotency, differentiation potential and karyotype stability. Gene expression profiling reveals novel insights into the adaption of hPSCs to continuous 3D culture compared to conventional 2D controls.

**Conclusions:**

Together, an entirely matrix-free, highly efficient, flexible and automation-friendly hPSC expansion strategy is demonstrated, facilitating the development of good manufacturing practice-compliant closed-system manufacturing in large scale.

**Supplementary Information:**

The online version contains supplementary material available at 10.1186/s13287-024-03699-z.

## Background

Due to their inherent capacity of unlimited proliferation and lineage differentiation, hPSCs have a great potential for advancing drug screenings, for modelling developmental processes and diseases, and for fueling cell-based regenerative therapies [1].

Recently, an increasing number of clinical trials based on hPSC-derived progenies has been initiated [2]. However, depending on the target therapy, estimated cell doses per patient range from 10^9^ to 10^10^ cells [3, 4]. These estimations emphasize that the envisioned routine application of hPSC-based therapies will require large cell quantities to be generated by robust and economically viable production processes, compatible with clinical and regulatory standards. Evidently, approaches relying on conventional two-dimensional (2D) culture conditions currently dominating the cell supply for first-in-human studies [5] are insufficient to meet the cell demands for routine hPSC-based therapies to thousands of patients in the foreseeable future [6, 7].

Previously, instrumented stirred tank bioreactor (STBR) systems, which are covering a wide range of process scales and are well established in mammalian bioprocessing [8], have been adapted to the matrix-free suspension culture of hPSCs as cell-only aggregates [9–12]. The STBR technology enables a homogeneous cultivation environment while key process parameters such as pH, dissolved oxygen concentration (DO), the rate of media perfusion and other conditions can be monitored and controlled [4]. Applying and optimizing these parameters recently enabled the advanced high density bioprocessing of hPSCs by metabolic control and in silico modelling, yielding up to 35 × 10^6^ cells/mL in a culture scale of 150 mL, which is equivalent to a 70-fold expansion of the inoculated hPSCs within 7 days [13, 14]. However, the applicability of this protocol to clinically relevant process dimensions and to specific GMP requirements is currently limited by the necessity to pre-expand the hPSCs in 2D adherent culture for subsequent bioreactor inoculation in 3D suspension culture.

To further close the gap between the current research state and industry requirements, process upscaling in 3D has been demonstrated by serial passaging, that is by dissociating the hPSC aggregates formed in suspension culture into single cells between each passaging step [15–17], but a number of key limitations remain. The published serial passaging processes are rather inefficient regarding the observed cell yield. This raises the question whether prolonged entirely matrix-free suspension culture is capable of maintaining hPSC properties, in particular if the 3D process is directly inoculated with cryopreserved cells, thereby completely rendering the necessity for matrix-dependent 2D pre-cultures.

The application of biocompatible extracellular matrix (ECM) scaffolds combined with biomimetic microcarriers has been suggested as alternative strategy for suspension culture transition of hPS cell cultivation [18]. However, microcarriers and matrices represent additional components to the process that must be validated and potentially removed from the final cell product during the downstream processing, adding extra hurdles and costs [6, 18].

All these challenges in mind, we here focused on advancing our recent protocol for the efficient high density bioprocessing of hPS cells-only aggregates in suspension towards serial passaging, thereby also mimicking a “seed train approach”, typically applied for the stepwise process upscaling of conventional mammalian cell lines in industry [19, 20]. For the comprehensive multi-parametric process assessment along extensive serial passaging, we have monitored: hPSCs’ growth and aggregation kinetics, maintenance of pluripotency and differentiation potential, karyotype stability, metabolic aspects, gene expression patterns and others. Moreover, different cryopreservation conditions of relatively large cell stocks were tested in-between the serial passaging approach, which is of key importance to ensure entire process transition to full GMP. As a result, we demonstrate matrix-free high-density bioprocessing of hPSCs promoting straightforward upscaling and GMP-compliance.

## Methods

### Adherent hPSC culture in monolayer

For all optimization experiments the hiPS cell line hHSC_1285 (HSC1285_T-iPS2; MHHi006-A) [21] derived from hematopoietic stem cells was used. The hiPS cell line GMPDU_8 (CD34 + hPBHSC_GMPDU_SeV-iPS8; MHHi008-A) [22] was used for validation of the multiple passage suspension culture approach. For the initial bioreactor inoculation, conventional 2D cell culture was performed as described [14]. In brief, cells were cultivated on Geltrex (Thermo Fisher Scientific)-coated flasks at feeder-free conditions. For the first 48 h, cells were cultivated in Essential 8 medium (E8; Additional file 1; Table S1) supplemented with 10 µM Y-27632 (RI; Tocris Bioscience) and thereafter in E8 only. Cells were passaged strictly every 72 h and cultivated for a total of 3 passages prior to bioreactor cultivation.

### Bioreactor cultivation and process parameters

For experiments a DASbox Mini Bioreactor System and a DASGIP Bioblock (Eppendorf SE) were used, which are both compact four-fold systems for parallel operation of four or more independently controllable bioreactor glass vessels for cell culture applications equipped with an eight-blade impeller (60° pitch) optimized for hPSC expansion [11]. For media perfusion a porous outflow filter (20- to 40-mm pore size) was incorporated as a cell retention device similar to that described by Weegman et al. [23]. Calibration of pumps and sensors including other basic culture conditions for stirred suspension culture was performed as described elsewhere [14]. In brief, bioreactors were inoculated at a viable cell density of 5 × 10^5^ cells/mL in E8 + RI + Pluronic F-68 (Thermo Fisher Scientific) in a total volume of 150 mL (DASbox Mini Bioreactor System) or 500 mL (DASGIP Bioblock) and cells were cultivated at 37 °C. The suspension was stirred at 80 rpm in the DASbox and 61 rpm in the Bioblock, while headspace aeration was set to 0.9 sL/h (DASbox) or 3 sL/h (Bioblock) with 21% O_2_ and 5% CO_2_. After 24 h, medium was exchanged via perfusion at a successively increasing rate (Additional file 1; Table S2), with additional glucose and glutamine supplementation from day 1 onwards (Additional file 1; Table S1). The control of pH was initiated at a set point of 7.1, initially by reducing CO_2_ in the gas stream and afterwards by the addition of 1 M NaHCO_3_ (Merck). The DO probe was calibrated at 100% air saturation and DO was controlled at a set point of 40%.

### Chemical aggregate dissociation for cell passaging

For cell/aggregate passaging, stirring was interrupted to let the aggregates settle by gravity for ≈3 min. To avoid cell loss, the expended medium was thoroughly removed through the inoculation port, transferred into the inoculation bottle (Additional file 1; Figure S1; applying negative pressure to the bottle by a conventional pipetting device), and discarded by pipetting from the bottle. Next, Phosphate Buffered Saline without Ca^2+^/Mg^2+^ (PBS w/o; Thermo Fisher Scientific; 150 mL for the DASbox and 500 mL for the Bioblock) was added to the bottle and transferred into the bioreactor for aggregate washing. After aggregate settling for ≈3 min, PBS w/o was removed from the reactor as described above. For aggregate dissociation, pre-warmed Versene solution (Thermo Fisher Scientific; 100 mL for DASbox and 250 mL for Bioblock) was added to the bioreactor via the incoulation bottle procedure: this is performed in two steps each applying half of the total Versene volume, to ensure back-flushing of any aggregates that might have collected in the tubing connecting the bioreactor with the inoculation bottle. For promoting controlled dissociation, stirring at 120 rpm (DASbox) or 80 rpm (Bioblock) and 37 °C was applied for 10–15 min. Next, an equal amount of DMEM/F-12 (Thermo Fisher Scientific) matching the Versene volume was added (via inoculation bottle), to quench/stop the dissociation reaction. The resulting cell suspension was collected in the inoculation bottle and transferred into a conical 500 mL tube (Corning) for centrifugation at 300×*g* and 4 °C for 3 min. The supernatant was aspirated and the cells were re-suspended in E8 + RI: 50 mL for DASbox; 100 mL for Bioblock. In a parallel procedure, E8 + RI (100 mL DASbox; 350 mL Bioblock) is pre-warmed in the reactor vessel for process re-inoculation. The harvested hPSCs were counted as described earlier [14] and re-inoculated at 5 × 10^5^ cells/mL at the respective final volume (150 mL DASbox; 500 mL Bioblock).

### Cells and media sampling

For the parallel cell- and media- sampling from the bioreactors, 3 mL samples were collected every 24 h (plus direct sampling after inoculation) without interruption of stirring, as described [14]. For the analysis of aggregate morphology and diameter, at least 3 independent light microscopic pictures were captured per sample and the diameter was automatically determined via an ImageJ based macro [13, 24]. Mean diameters represent the arithmetic average of > 900 single aggregates. For cell counting and analysis, aggregates were dissociated into single cells with Accutase (Merck) for cell counting, cell cycle analysis and flow cytometry as described [14]. The media supernatant was stored at − 20 °C for subsequent analysis of glucose/lactate concentrations via Biosen C Line (EKF Diagnostics) and medium osmolality via Osmomat 3000 (Gonotec) according to manufacturer’s instruction. Moreover, single cells were analyzed for cell cycle phase distribution and expression of markers related to an undifferentiated state via flow cytometry.

### Cell cycle analysis

For cell cycle analysis, aggregates were dissociated and ≈5 × 10^5^ cells were re-suspended with 70% (v/v) ethanol (Th. Geyer) in PBS w/o and stored at −20 °C until used. For analysis, cells were washed with PBS w/o, permeabilized with 0.1% (v/v) Triton X-100 (Merck) and additionally stained for 5 min with 1.7 μg/mL of 40,6-diamidino-2-phenylindole (DAPI; Merck) at 37 °C. Afterwards, cells were washed with and re-suspended in PBS w/o and analysis was performed with the MACSQuant Analyzer 10 Flow Cytometer (Miltenyi Biotec) and the FlowJo v10 software (FlowJo, LLC).

### Flow cytometry analysis of markers associated with an undifferentiated state

Surface markers associated with an undifferentiated state SSEA-3, SSEA-4 and TRA-1–60 were analyzed via triple staining of ≈1.5 × 10^5^ single cells with respective antibodies by incubation for 12 min at RT. Antibodies were diluted in PBS w/o.

For the analysis of intracellular markers OCT-3/4 and NANOG as well as the proliferation marker KI67, ≈1.5 × 10^5^ single cells were fixed with 1% (w/v) paraformaldehyde (PFA; Merck) at RT for 15 min. After washing with PBS w/o, cells were incubated with antibodies against NANOG + OCT-3/4 in a double staining and KI67 in a single staining for 12 min at RT. Antibodies were diluted in a 1:1 ratio of PBS w/o and FIX&PERM Solution B (Nordic-MUbio BV).

After staining, cells were washed with and re-suspendend in PBS w/o and analysis was performed with the MACSQuant Analyzer 10 Flow Cytometer and the FlowJo v10 software. Respective antibodies are listed in Table S3 (Additional file 1).

### RNA isolation and transcriptional analysis

For RNA isolation, process-derived aggregates were dissociated into single cells (as described above). Thereafter ≈3 × 10^6^ cells were treated with 500 µL TRIzol® reagent (Thermo Fisher Scientific), mechanically disrupted by pipetting and stored at −80 °C until further processed. After thawing, 100 µL chloroform (Merck) was added and the mixture was centrifuged at 12,000×*g* and 4 °C for 15 min. Afterwards, the RNA-containing aqueous phase was separated and RNA was prepared using the NucleoSpin RNA Kit II (Macherey–Nagel) according to manufacturer’s instruction.

RNA samples with a concentration of 500–1900 ng/µL were submitted to the Research Core Unit Genomics (RCUG) of the Hannover Medical School (MHH) for quality control and bulk RNA sequencing. Normalized counts (provided by RCUG) served as input for further analysis of significant (*p* < 0.05) differentially expressed genes (DEGs) with the Qlucore Omics Explorer 3.8 software. Gene set enrichment analysis was performed with the web-based tool Enrichr [25, 26].

### Cryopreservation of suspension-derived single cells for direct bioreactor inoculation

For cryopreservation experiments, Bioblock-derived hPS cells generated by above described dissociation procedure were used. E8 + RI-suspended cells were counted and the respective amount of cells was transferred into a 15 mL conical tube (Greiner AG) for subsequent centrifugation at 300×*g* and 4 °C for 3 min. Collected cells were re-suspended in a respective amount of cryopreservation medium consisting of 90% E8, 10% dimethyl sulfoxide (DMSO; Merck), 0.1% Pluronic F-68 (Thermo Fisher Scientific) and 10 µM RI (Tocris Bioscience) to achieve the desired cell concentration followed by aliquoting of 1 mL into CRYO.S freezing tubes (Greiner AG). Cryopreservation was performed with a Planer Kryo 10 Series III controlled-rate freezer (Planer Limited) with a defined protocol (Additional file 1; Table S4) followed by long-term storage at −150 °C. Cells have been cryopreserved for at least 3 weeks before further cultivation. For direct suspension culture inoculation in bioreactors, cells were thawed in a water bath at 37 °C for ≈3 min, diluted in cooled 10 mL of E8 + RI and collected by centrifugation at 300 × *g* and 4 °C for 3 min for re-suspension in 20 mL E8 + RI followed by cell counting. Bioreactors were inoculated as described above at the established density of 5 × 10^5^ cells/mL.

### Directed cardiomyogenic differentiation

For chemically defined, directed cardiac differentiation in the DASbox, aggregates were dissociated at the end of respective process conditions and single cells were re-inoculated at 5 × 10^5^ cells/mL at 150 mL. After three days of culture in E8, the cell density was adapted to 5 × 10^5^ cells/mL at 150 mL (based on the analysis of aggregate samples) and cardiac differentiation of the pre-formed aggregates was performed as described [24, 27]. In brief, cells were cultivated in CDM3 (Table S5) [28] supplemented with 5 μM CHIR99021 (Institute for Organic Chemistry, Leibniz University Hannover). After 24 h, medium was switched to CDM3 supplemented with 5 μM IWP2 (Tocris Bioscience). After another 48 h, medium was switched to CDM3 only and exchanged every 2–3 days thereafter. Analysis of cardiomyocyte-specific markers was performed on day 10 after CHIR-induction.

### Flow cytometry analysis of cardiomyocyte-specific markers

For analysis of cardiomyocyte-associated markers, cardiac aggregates were dissociated at differentiation day 10 using the STEMdiff dissociation Kit (STEMCELL Technologies). Aggregates were incubated in 1 mL of STEMdiff enzyme solution in a water bath at 37 °C for 5–8 min while gently shaking to support dissociation. The obtained single cardiomyocytes were re-suspended in 5 mL of STEMdiff support medium and then centrifuged at 300×*g* and 4 °C for 3 min. The supernatant was removed and cells were re-suspended in PBS w/o.

For staining, ≈1.5 × 10^5^ cells were fixed with 1:1 diluted FIX&PERM Solution A (Nordic-MUbio BV) with PBS w/o for 10 min at RT. After washing with PBS w/o, cells were incubated with respective primary antibodies (diluted in a 1:1 ratio of FIX&PERM Solution B and PBS w/o) for 45 min at RT. After another washing step with PBS w/o, cells were incubated with appropriate secondary antibodies (diluted in a 1:1 ratio of FIX&PERM Solution B and PBS w/o) for 25 min at 4 °C. Following a final washing step with PBS w/o, cells were re-suspended in PBS w/o and analyzed with the MACSQuant Analyzer 10 Flow Cytometer the FlowJo v10 software. Respective antibodies are listed in Table S6 (Additional file 1).

### Undirected germ layer differentiation

Undirected differentiation for testing of unrestricted germ layer differentiation was performed as described [29]. In brief, aggregates were harvested from the reactor at the respective process endpoint and cultivated in undirected differentiation medium (for composition see Additional file 1; Table S7) in ultra-low attachment 6-well plates (Greiner AG) for 7 days at ≈1 × 10^6^ cells/mL in 3 mL per well. Subsequently, cells were transferred to gelatin-coated 12-well plates (Greiner AG) with 1 mL of medium per well for another 14 days. Medium was replaced every 2 days. Lineage analysis was performed via immunocytological staining.

### Immunocytological staining of pluripotent and undirected differentiated cells

Cells were fixed with 4% (w/v) PFA at RT for 10 min. After washing the cells with PBS w/o followed blocking with 5% (w/v) donkey serum (Merck) and 0.25% (v/v) Triton X-100 (Merck) in Tris-Buffered Saline (TBS; Table S8) for 20 min at RT. Then cells were incubated with respective primary antibodies diluted in staining buffer (TBS containing 1% (w/v) BSA; Merck) at 4 °C overnight. Then cells were washed with TBS and incubated with secondary antibodies for 30 min at RT. Next, after washing with TBS, cells were counterstained with 1.7 μg/mL DAPI for 15 min at RT, covered with PBS containing Ca^2+^/Mg^2+^ (PBS+ ; Thermo Fisher Scientific) and analyzed via Axio Oberserver A1 fluorescence microscope and Axiovision software (Zeiss). Respective antibodies are listed in Table S9 (Additional file 1).

### Cryosectioning and immunocytological staining of hPSC aggregates

At process endpoint, hPSC aggregates were harvested through the inoculation port and cryoembedded in Tissue-Tek (Sakura Finetek) in respective cryomolds (Sakura Finetek) utilizing a Microm HM 560 cryotome (Thermo Fisher Scientific). Frozen hPSC aggregates were stored at − 80 °C until tissue sections of 10 µm in thickness were generated using the Microm HM 560 cryotome, dried at RT overnight and stored at − 80 °C until use.

For immunocytological staining, cryosections were fixed with 4% (w/v) PFA for 5 min at RT. After washing with TBS followed blocking with 5% (w/v) donkey serum and 0.25% (v/v) Triton X-100 (Merck) in TBS for 1 h at RT and overnight incubation with primary antibodies diluted in staining buffer (TBS containing 1% (w/v) BSA) at 4 °C. Next, cryosections were washed with TBS and stained with secondary antibodies diluted in staining buffer for 1 h at RT. Cryosections were washed with TBS and counterstained with 1.7 μg/mL DAPI for 15 min at RT, washed with TBS and then mounted with mounting medium (Agilent Technologies). The cryosections were kept at RT overnight before analysis with the Axio Oberserver A1 fluorescence microscope and Axiovision software (Zeiss). Respective antibodies are listed in Table S10 (Additional file 1).

### Calculation of the specific growth rate µ

The specific growth rate µ [d^−1^] is calculated as

$$\mu = \left( {\frac{{{\text{X}}_{{{\text{t}}_{{{\text{n}} + {1}}} }} - {\text{X}}_{{{\text{t}}_{{\text{n}}} }} }}{{{\text{t}}_{{{\text{n}} + {1}}} - {\text{t}}_{{\text{n}}} }}} \right) \times \frac{{1}}{{{\overline{\text{X}}}_{{{\text{t}}_{{{\text{n}} + {1}}} }} }}$$

with X being the viable cell density [cells/L] at a given time point t [d] and the mean viable cell concentration ${\overline{\text{X}}}$ calculated as

$${\overline{\text{X}}}_{{{\text{t}}_{{{\text{n}} + {1}}} }} = \frac{{{\text{X}}_{{{\text{t}}_{{{\text{n}} + {1}}} }} - {\text{X}}_{{{\text{t}}_{{\text{n}}} }} }}{{{\text{ln}}\left( {{\text{X}}_{{{\text{t}}_{{{\text{n}} + {1}}} }} } \right) - {\text{ln(X}}_{{{\text{t}}_{{\text{n}}} }} {)}}}$$

### Calculation of the cell-specific glucose consumption and lactate production rate

The cell-specific glucose consumption rate qGlc [pmol/(cell × d)] for the process days without medium change was calculated as

$${\text{qGlc}}_{{{\text{t}}_{{{\text{n}} + {1}}} }} = - \left( {\frac{{{\text{Glc}}_{{{\text{t}}_{{\text{n + 1}}} }} - {\text{Glc}}_{{{\text{t}}_{{\text{n}}} }} }}{{{\text{t}}_{{\text{n + 1}}} - {\text{t}}_{{\text{n}}} }}} \right) \times \frac{{1}}{{{\overline{\text{X}}}_{{{\text{t}}_{{{\text{n}} + {1}}} }} }}$$

with Glc being the glucose concentration [pmol/L] and t the respective time point [d]. In the perfused cultures the cell-specific glucose consumption rate was calculated as

$${\text{qGlc}}_{{{\text{t}}_{{{\text{n}} + {1}}} }} = - \left[ {\left( {\frac{{{\text{Glc}}_{{{\text{t}}_{{{\text{n}} + {1}}} }} { } - {\text{Glc}}_{{{\text{t}}_{{\text{n}}} }} }}{{{\text{t}}_{{{\text{n}} + {1}}} { } - {\text{t}}_{{\text{n}}} }}} \right) + \frac{{\text{F}}}{{\text{V}}}\left( {\overline{{{\text{Glc}}}}_{{{\text{t}}_{{{\text{n}} + {1}}} }} - {\text{Glc}}_{{\text{f}}} } \right)} \right]{ } \times \frac{{1}}{{{\overline{\text{X}}}_{{{\text{t}}_{{{\text{n}} + {1}}} }} }}$$

with F being the perfusion rate [L/d], V the culture volume [L], Glc_f_ the glucose concentration in the feed stream [pmol/L] and $\overline{{{\text{Glc}}}}$ the mean glucose concentration in the culture calculated as

$$\overline{{{\text{Glc}}}}_{{{\text{t}}_{{{\text{n}} + {1}}} }} = \frac{{{\text{Glc}}_{{{\text{t}}_{{{\text{n}} + {1}}} }} - {\text{Glc}}_{{{\text{t}}_{{\text{n}}} }} }}{{{\text{ln}}\left( {{\text{Glc}}_{{{\text{t}}_{{{\text{n}} + {1}}} }} } \right) - {\text{ln}}\left( {{\text{Glc}}_{{{\text{t}}_{{\text{n}}} }} } \right)}}$$

The cell-specific lactate production rate qLac [pmol/(cell × d)] was similarly calculated as

$${\text{qLac}}_{{{\text{t}}_{{{\text{n}} + {1}}} }} = \left( {\frac{{{\text{Lac}}_{{{\text{t}}_{{{\text{n}} + {1}}} }} - {\text{Lac}}_{{{\text{t}}_{{\text{n}}} }} }}{{{\text{t}}_{{{\text{n}} + {1}}} - {\text{t}}_{{\text{n}}} }}} \right) \times \frac{{1}}{{{\overline{\text{X}}}_{{{\text{t}}_{{{\text{n}} + {1}}} }} }}$$

with the lactate concentration Lac [pmol/L]. In the perfused culture, the cell-specific lactate production rate is calculated as

$${\text{qLac}}_{{{\text{t}}_{{{\text{n}} + {1}}} }} = \left[ {\left( {\frac{{{\text{Lac}}_{{{\text{t}}_{{{\text{n}} + {1}}} }} - {\text{Lac}}_{{{\text{t}}_{{\text{n}}} }} }}{{{\text{t}}_{{{\text{n}} + {1}}} - {\text{t}}_{{\text{n}}} }}} \right) + \frac{{\text{F}}}{{\text{V}}}\overline{{{\text{Lac}}}}_{{{\text{t}}_{{{\text{n}} + {1}}} }} } \right]{ } \times { }\frac{{1}}{{{\overline{\text{X}}}_{{{\text{t}}_{{{\text{n}} + {1}}} }} }}$$

where $\overline{{{\text{Lac}}}}$ is the mean lactate concentration in the culture calculated as

$$\overline{{{\text{Lac}}}}_{{{\text{t}}_{{{\text{n}} + {1}}} }} = \frac{{{\text{Lac}}_{{{\text{t}}_{{{\text{n}} + {1}}} }} - {\text{Lac}}_{{{\text{t}}_{{\text{n}}} }} }}{{{\text{ln}}\left( {{\text{Lac}}_{{{\text{t}}_{{{\text{n}} + {1}}} }} } \right) - {\text{ln}}\left( {{\text{Lac}}_{{{\text{t}}_{{\text{n}}} }} } \right)}}$$

The yield coefficient Y of lactate from glucose was calculated as

$${\text{Y}}\left( {\text{qLac/qGlc}} \right) = \left| {\frac{{{\text{qLac}}}}{{{\text{qGlc}}}}} \right|$$

### Statistical analysis

All bioreactor runs were performed in at least three independent biological replicates. Unless otherwise indicated, statistical analyses were performed with GraphPad Prism 7.04 (GraphPad Software Inc.). Data is presented as mean ± standard error of the mean (SEM). All experiments were performed in at least three biological replicates. Technical replicates for independent measurements or assays were performed at least twice. Statistical significance was determined by ordinary one-way analysis of variance (ANOVA) or two-way ANOVA followed by Turkey's post-test and was assigned as *p* < 0.05 (*), *p* < 0.01 (**), *p* < 0.001 (***) and *p* < 0.0001 (****).

## Results

### Time restricted hPSC aggregate culture promotes maintenance of maximum growth kinetics

**Fig. 1:**
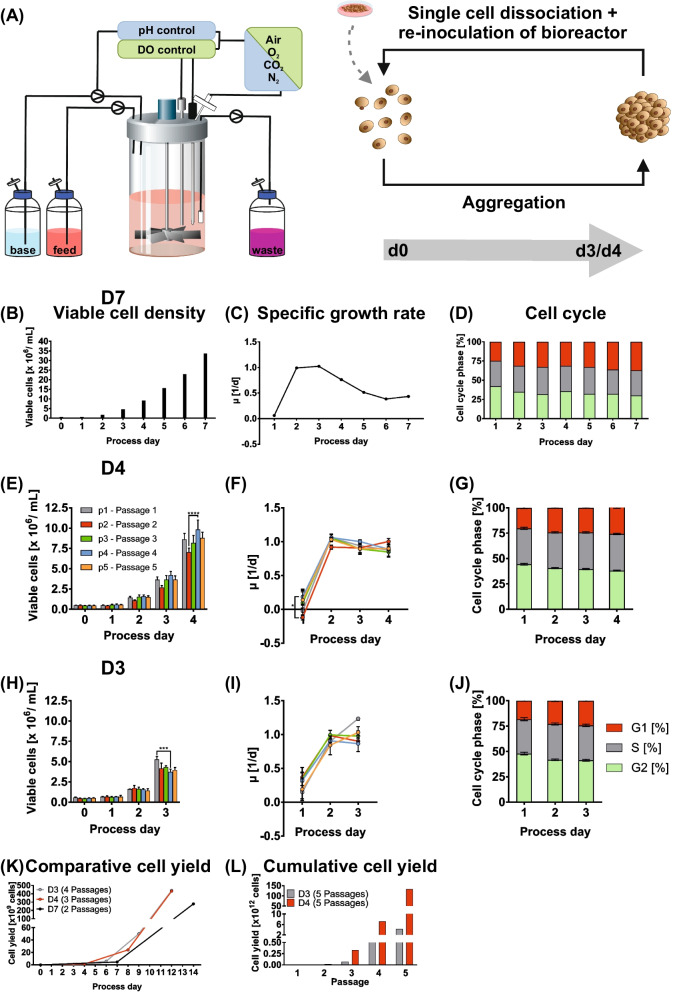
Influence of cultivation interval on growth kinetics and cell cycle distribution. **A** Adherent 2D culture-derived human pluripotent stem cells (hPSCs; hHSC_1285) were once detached and seeded as single cells at 5 × 10^5^ cells/mL to stirred tank bioreactors (STBRs). After 24 h, cultivation medium was exchanged via perfusion, while a porous glass filter was installed to retain hPSC aggregates inside the bioreactor. Throughout the culture, pH and dissolved oxygen concentration (DO) were controlled via triggered clock. The pH level was controlled to 7.1 initially by reduction of CO_2_ in the gas supply and afterwards by addition of 1 M NaHCO_3_. The DO was similarly regulated at 40% (of air saturation) by adaption of the O_2_ concentration in the supply gas. In two separate cultures, hPSC aggregates were dissociated inside the bioreactor after 3 or 4 process days and single cells were seeded back into the bioreactor at 5 × 10^5^ cells/mL. This was repeated for a total of 5 passages, resulting in the consecutive passages p1 (grey), p2 (red), p3 (green), p4 (blue) and p5 (orange) (n = 3–11). **B**–**D** Exemplary viable cell density, specific growth rate µ and cell cycle analysis for the 7 day lasting historic process (D7) averaged for three independent cell lines. **E**–**J** Viable cell density, specific growth rate µ and cell cycle analysis (5 passage average) for a cultivation interval of 4 days (D4; **E**–**G**) and 3 days (D3; **H**–**J**). **K** Comparison of cell yield between the historic process (D7, black) and the shortened processes of 3 days (D3, grey) and 4 days (D4, red). Cumulative cell yield was calculated based on average fold expansions. **L** Cumulative cell yield for a total of 5 passages calculated based on average fold expansion for each individual passage in both approaches D3 and D4. Results are presented as mean ± standard error fo the mean (SEM)

For bioreactor inoculation at ≈0.5 × 10^6^ single cells/mL, hPSCs were initially expanded by 2D monolayer culture (schematic Fig. 1A). Applying perfusion feeding (including stepwise adaptation of the media throughput detailed in Methods), pH and the DO was feedback-controlled as recently established [13, 14]. On process day 7 (d7) about 35 × 10^6^ cells/mL were observed for all three independent cell lines tested (Fig. 1B). A maximum specific growth rate of ≈1.0 d^−1^ was found on d2–d3, progressively declining to ≈0.76 d^−1^ from d4 onwards (Fig. 1C). Parallel cell cycle analysis revealed a low but progressive increase of cells in the G1 phase (indicative of non-proliferating resting cells) from ≈25% on d0 to ≈37% on d7 (Fig. 1D).

This dataset indicates an optimal hPSC proliferation at our culture conditions until d3 – d4 only, suggesting further potential for process improvement by shortening the process duration from previously established 7 days (process designated as D7) [10, 11, 13] to 3 or 4 days (designated D3 and D4, respectively). Thus, we have next tested the dissociation of suspension-derived hPSC aggregates on day 3 or 4, respectively, followed by “cyclic suspension culture re-inoculation” of single cells seeded at ≈5 × 10^5^ cells/mL.

Notably, in contrast to previously published enzymatic dissociation of suspension culture-derived hPSC aggregates [16, 17], we here established chemical hPSC aggregate dissociation directly in the bioreactor vessel under controlled temperature and impeller-based stirring at 120 rpm. For the sufficient dissociation of D3 (Additional file 1; Figure S2B) and D4 (Additional file 1; Figure S2C) aggregates into single cells, ≈12 min of treatment were required, resulting in cell suspensions equivalent to the enzymatic detachment of hPSCs from 2D monolayer (Additional file 1; Figure S2A).

The dissociation/re-inoculation cycle was repeated for 4 times, resulting in 5 sequential passages (including the first passage inoculated by 2D monolayer-derived hPSCs). In the first suspension passage (p1) of the D4 approach, an average cell density of 8.1 × 10^6^ cells/mL was reached on d4, closely recapitulating the 9.1 × 10^6^ cells/mL observed on d4 at previous D7 conditions. The following passages yielded similar cell densities of 6.6 (p2), 7.9 (p3), 9.7 (p4) and 9.2 × 10^6^ cells/mL (p5), while the reduced value after p2 was noted (Fig. 1E). However, calculations of the specific growth rate revealed equal values and patterns of growth kinetics for all 5 passages tested, showing a maximum of 0.9–1.1 d^−1^ around d2–d3 (Fig. 1F). In accordance with D7 process data, a slight increase of a resting cell population in G1 from ≈20% (d1) to ≈26% (d4) was observed for all 5 passages tested (Fig. 1G), creating a striking “reset pattern” after single cell re-inoculation for all passages (Additional file 1; Figure S3B).

For D3 conditions, a maximum cell density of 5.2 × 10^6^ cells/mL was found on d3 in p1, in accordance with the 4.6 × 10^6^ cells/mL reached on d3 in D7. Similar but slightly lower cell densities of 4.2 (p2), 4.3 (p3), 3.7 (p4) and 4.0 × 10^6^ cells/mL (p5) were observed in subsequent passages (Fig. 1H). In p1, the specific growth rate on d2–d3 was at 0.9–1.2 d^−1^ (equivalent to D4 and D7 conditions), remaining high at 0.9–1.0 d^−1^ on d2–d3 in following passages (Fig. 1I). The cell cycle pattern in D3 was similar to D4 and D7, while the population of resting cells in G1 notably increased from ≈18% on d1 to ≈24% on d3 (Fig. 1J), thereby again reflecting a cyclic “reset pattern” (Additional file 1; Figure S3A).

We subsequently compared the D3 and D4 output to our former D7 approach (Fig. 1K). Calculating the cumulative yield for 12 days, D3 yielded 4.37 × 10^11^ cells (i.e. in 4 passages), while D4 yielded 4.53 × 10^11^ cells (in 3 passages). To simplify the comparison to the D7 approach, the output of 2 passages was summated, resulting in 2.79 × 10^11^ cells in 14 days. An additional calculation for the 5 passages tested revealed that the D3 approach generated a cumulative cell yield of 3.76 × 10^12^ cells in 15 days, whereas the D4 strategy yielded 1.29 × 10^14^ cells in 5 passages equivalent to 20 days (Fig. 1L).

Overall, D3 and D4 share great similarity in maintaining the aspired maximum growth kinetics of hPSCs. This is supported by process-specific profiles of pH and DO revealing both high reproducibility across the 5 passages tested for each culture strategy and the cross-comparison of D3 versus D4 as well (Additional file 1; Figure S3C, D). Moreover, osmolality profiles of the culture medium, which increases as a direct result of base addition to maintain pH control (Additional file 1; Figure S3E, F), as well as the oxygen concentration pattern XO_2_ in the supply gas adapted for DO control (Additional file 1; Figure S3G, H) also share similar characteristics between the different passaging intervals tested.

### hPSCs’ aggregate patterning and metabolic activity is maintained in matrix-free long-term suspension culture independent of the passaging interval

**Fig. 2:**
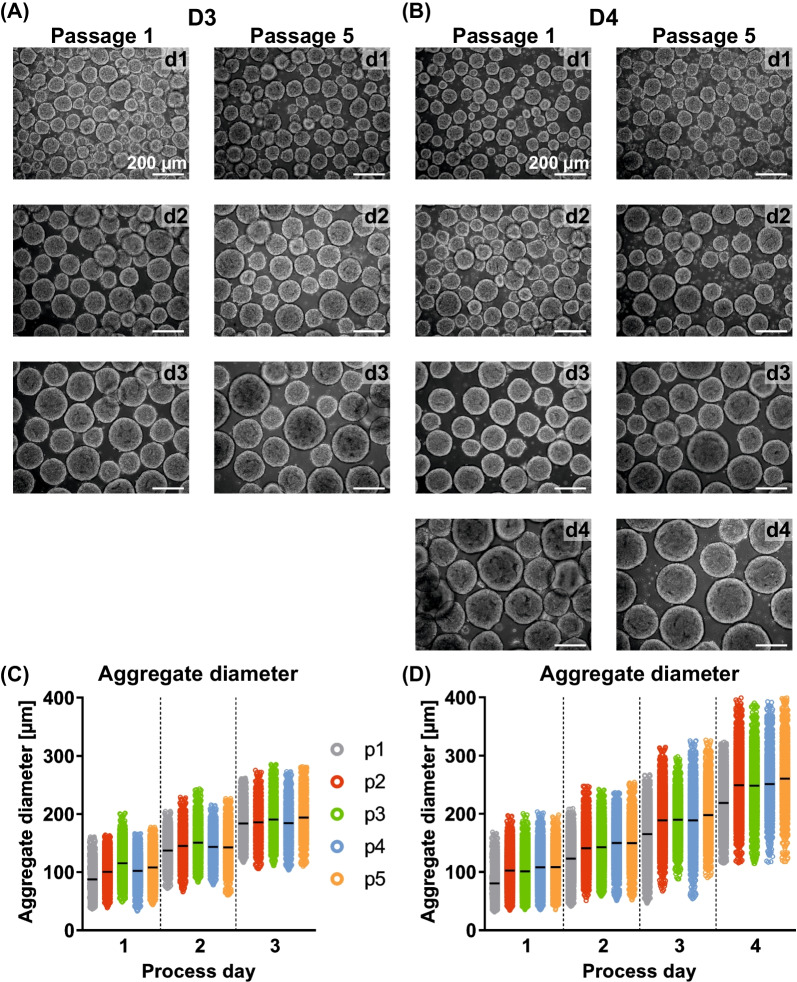
Influence of cultivation interval on aggregate size distribution and morpholoy. **A**, **B** Exemplary light microscopic pictures of process-derived aggregate samples on all process days of passage 1 and passage 5 for the 3 day (D3) and the 4 day (D4) cultivation interval (scale bar = 200 µm). **C**, **D** Aggregate diameter distribution over the cultivation time for D3 and D4 in consecutive passages p1 (grey), p2 (red), p3 (green), p4 (blue) and p5 (orange) (n = 3–11)

Continuous passaging of suspension-cultured hPSCs did not induce apparent changes in aggregate morphology and aggregate size patterning after repeated single cell inoculation. Throughout the 5 passages tested, aggregates maintained a smooth, round morphology in D3 and D4 (Fig. 2A, B). On d1, the mean aggregate diameter ranges at 80–120 µm for both conditions (Fig. 2C, D). Equivalently, on day 3, the mean aggregate diameter ranges at 185–195 µm in D3 and 170–200 µm in D4, indicating highly similar aggregate growth kinetics independent of the cultivation interval. Aggregates in D4 reached a maximum mean diameter of 220–260 µm on d4, which is notably below the critical 300 µm level reported to be potentially growth inhibiting due to diffusion limits [30].

Sufficient glucose supply for avoiding cell starvation has been reported critical for maximizing the cell yield [13]. Starting with a glucose concentration of 16–17 mM measured after fresh E8 addition, the values readily decrease on d1 to 8.5–11 mM in D3 (Additional file 1; Figure S4A) and 10–13 mM in D4, respectively (Additional file 1; Figure S4B). Subsequently, perfusion with glucose-enriched culture medium was applied, resulting in stabilized glucose concentrations ranging at 14–19 mM (Additional file 1; Figure S4A, B). Correspondingly, lactate concentrations constantly increased to 22.5–25.5 mM on d3 in D3 (Additional file 1; Figure S4C) and 21–26.5 mM on d4 in D4 (Additional file 1; Figure S4D), showing comparable patterns for all 5 passages.

We further calculated the cell-specific metabolite conversion rates to better monitor cells’ metabolism along the process. In both D3 and D4 (Additional file 1; Figure S4E-F), qGlc was ranging at 9–19 pmol/(cell × d) while peaking on d2, showing similar, almost overlapping profiles for all passages. This was closely matched by qLac profiles for D3 and D4, ranging at 11–25 pmol/(cell × d) with a maximum on d2. Finally, the yield coefficient calculated as a ratio of qLac and qGlc, was stably ranging at 1.3–1.5 on d2–d3 and d2–d4 (Additional file 1; Figure S4G-H), respectively, further confirming that hPSCs’ metabolism remained highly stable at least for the 5 passages of continuous, matrix-free suspension culture investigated.

### Gene expression patterning suggests 2D-to-3D-culture adaptation, but some differences among 3D samples are observable as well

Previous studies indicated that the transition from 2D adherent to 3D suspension culture may result in molecular changes in hPSCs [31, 32]. To investigate our approach, bulk gene expression patterning was performed on hHSC_1285 cells cultured in: 2D monolayer, harvested on d3 prior to bioreactor inoculation (sample termed: ML); 3D suspension, harvested from both the D3 and the D4 approach after earliest (p1) and latest (p5) passages on respective passage end points d3 (samples D3/p1 and D3/p5) or d4 (samples: D4/p1 and D4/p5).

Gene sets/pathways of DEGs comparing ML vs. 3D samples (D3/p1, D3/p5, D4/p1 and D4/p5; Additional file 1; Figure S5) with the web-based tool Enrichr [26] are listed in Table S11 (Additional file 1), revealing that pathways upregulated in ML correspond to *TGF-β* signaling (*LEFTY1*, *ID1*, *PITX2*, *THBS1*, *LEFTY2*, *BMP7*, *NODAL*, *SMAD7*), *PI3K/AKT* signaling (*PDGFRB*, *CDKN1A*, *ITGB5*, *ANGPT1*, *TNC*, *PDGFB*, *THBS1*, *FGF4*, *TCL1B*, *FGF8*, *LPAR6*, *COL4A3*, *TEK*) and heavy metal-binding metallothioneins (*MT2A*, *MT1F*, *MT1G*, *MT1X*, *MT1H*, *MMP9*, *MT1E*). In contrast, DEGs upregulated in 3D are predicted to be involved in cell adhesion (*CLDN11*, *NTNG2*, *MADCAM1*, *HLA-DQB1*), *AMPK* signaling (*FBP1*, *CFTR*, *CREB5*) and insulin secretion in response to glucose stimulus (*ADRA2A*, *CFTR*, *FOXA2*).

**Fig. 3:**
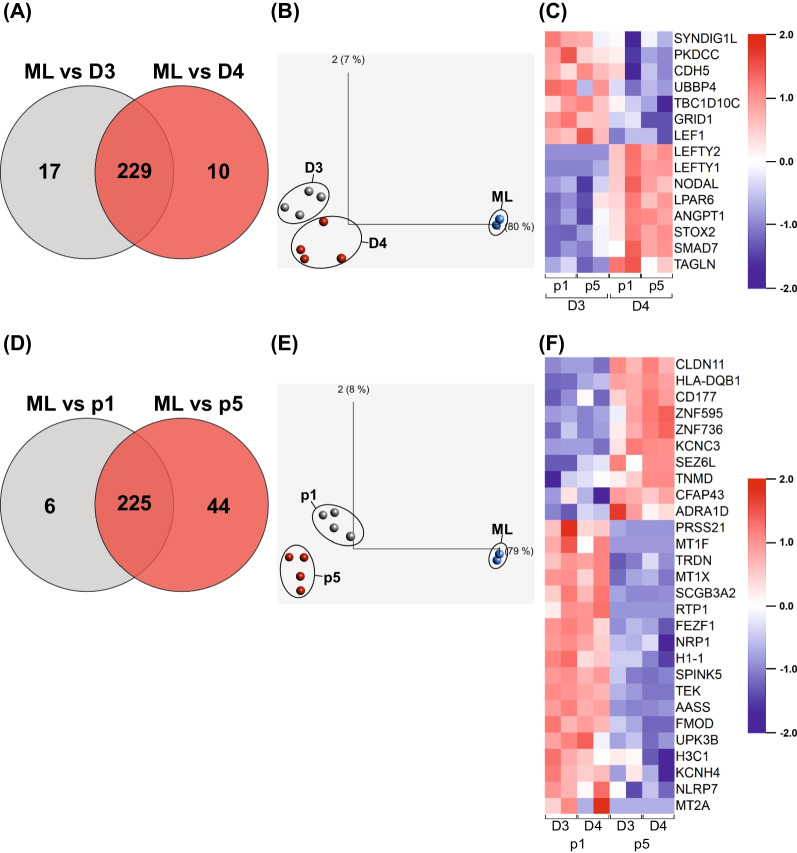
Gene expression analysis of 3D suspension culture-derived cell samples. **A** Venn diagram based on differentially expressed genes (DEGs) found between samples of ML and D3 or D4, respectively. **B** Principle component analysis (PCA) clustering samples according to D3 (grey), D4 (red) and ML (blue) origin. **C** Clustering and heat map analysis of significant (*p* < .05) DEGs beyond a twofold change threshold for D3 and D4-derived samples. **D** Venn diagram based on DEGs found between samples of ML and p1 or p5, respectively. **E** PCA clustering samples according to p1 (grey), p5 (red) and ML (blue) origin. **F** Clustering and heat map analysis of significant (*p* < .05) DEGs beyond a twofold change threshold for p1 and p5-derived samples

In more detail, a comparative analysis of ML versus 3D according to D3 and D4 origin revealed 256 significant (*p* < 0.05) DEGs (> twofold change; clustered in Additional file 1; Figure S6A), while 229 DEGs are shared between D3 and D4 (Fig. 3A). This high similarity of D3 and D4 is emphasized by their close clustering in the principle component analysis (PCA) compared to ML (Fig. 3B). Focused comparison of 3D according to the differential passaging intervals (D3 vs. D4) revealed 50 significant (*p* < 0.05) DEGs, 15 of which were beyond the twofold change threshold (clustered in Fig. 3C). DEGs upregulated in D3 are predicted to be involved in cell adhesion (*CDH5*, *NTNG2*, *MADCAM1*), *JAK/STAT* signaling (*PDGFRB*, *CDKN1A*, *THPO*) and kinase activity (*PDGFRB*, *PKDCC*, *TEK*; Figure S7A). On the other hand, DEGs upregulated in D4 correspond to *TGF-β* signaling and SMAD protein phosphorylation for regulation of pluripotency (*LEFTY1*, *LEFTY2*, *BMP7*, *NODAL*, *SMAD7*, *ID1*, *WNT3*; Additional file 1; Figure S7A).

A similar comparison of ML with 3D according to early (p1) and late passage (p5) origin revealed 275 significant (*p* < 0.05) DEGs (> twofold change; clustered in Additional file 1; Figure S6B), with 225 DEGs shared between p1 and p5, respectively (Fig. 3D); the p1 vs. p5 similarity is further emphasized by their close PCA clustering compared to ML samples (Fig. 3E). Among the 94 significant (*p* < 0.05) DEGs between p1 and p5, 28 genes were differentially expressed beyond a twofold change threshold (Fig. 3F). Gene set enrichment analysis for these DEG sets (Additional file 1; Figure S7B) reflects upregulation of DNA deamination-related pathways (*APOBEC3H*, *APOBEC3B*) in p5, whereas the pathways identified for p1 correspond to negative regulation of receptor signaling via the *JAK/STAT* pathway (*SOCS3*, *CAV1*, *MT1X*), metallothioneins (*MT2A*, *MT1F*, *MT1X*) and fluid shear stress (*CAV1*, *CAV2*, *PDGFB*, *CCL2*).

### Multiple passage suspension culture is fully compatible with hPSCs’ pluripotency, differentiation potential and karyotype stability

**Fig. 4:**
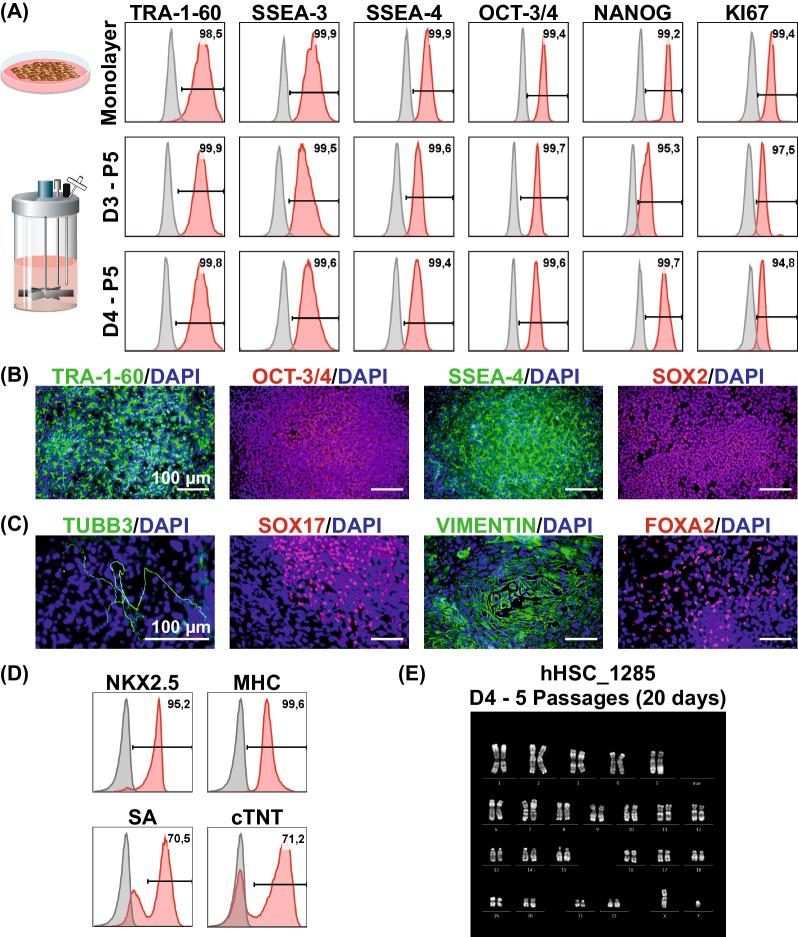
Influence of long-term, matrix-free suspension culture on pluripotency and genetic stability. **A** Exemplary flow cytometry analysis plots for surface markers associated with an undifferentiated state TRA-1-60, SSEA-3 and SSEA-4 as well as transcriptions factors OCT-3/4 and NANOG and proliferation marker KI67. Cells harvested at process endpoints of D3 (5 passages; 15 days) and D4 (5 passages; 20 days) were compared to monolayer-derived cells used for inoculation of the processes. **B** Representative immunocytological stainings of single cell-dissociated, seeded hPSC aggregates derived after 5 passages in D4 (20 days) stained for markers TRA-1-60, OCT-3/4, SSEA-4 and SOX2 (scale bar = 100 µm). **C** Undirected differentiation of D4 process-derived aggregates after 5 passages revealed expression of markers representing the three germ layers ectoderm (based on TUBB3), endoderm (based on SOX17 and FOXA2) and mesoderm (based on Vimentin). Scale bar = 100 µm. **D** Exemplary flow cytometry analysis plots of cardiomyocyte-specific markers NKX2.5, MHC, SA and cTNT after directed differentiation of D4-derived cells at the end of passage 5. **E** Karyotype of cells cultivated for 5 passages in D4 approach (20 days)

Pluripotency and unrestricted differentiation potential is a key feature of hPSCs. Analysis of markers associated with an undifferentiated state on the protein level revealed > 95% positivity for the transcription factors OCT-3/4, NANOG and the proliferation-associated marker KI67 as well as > 99% positivity for the surface markers TRA-1-60, SSEA-3 and SSEA-4 (Fig. 4A). These results were obtained after 5 passages of continuous suspension culture for both D3 (15 days) and D4 (20 days), thereby matching patterns of hPSCs that were initially detached from matrix-supported monolayer culture used for suspension culture inoculation. Aggregate-derived single cell dissociated hPSCs that were exemplarily seeded in monolayer after 5 passages of D4, showed robust nucleus-restricted expression of OCT-3/4 and SOX2 as well as surface markers TRA-1-60 and SSEA-4 in respective immunocytological staining (Fig. 4B).

Importantly, undirected differentiation of p5 aggregates from D4 resulted in the formation of cell lineages representing the three layers endoderm (SOX17, FOXA2), mesoderm (Vimentin) and ectoderm (TUBB3) shown in Fig. 4C. Moreover, the directed differentiation of such p5/D4 aggregates by an established protocol [24] resulted in the induction of functional cardiomyocytes at ≈70% purity (Fig. 4D). Lastly, in agreement with quality control guidelines [33], we analyzed the karyotype to exclude abnormalities in our matrix-free 3D approach, as exemplarily shown for the D4 process after 20 days of culture (Fig. 4E).

The D4 approach was repeated with the genetically independent hPSC line GMPDU_8 for 5 passages, closely reflecting hHSC_1285 results (Additional file 1: Figures S8, S9).

### Process upscaling and high density cryopreservation of suspension-derived hPSCs is compliant with direct bioreactor inoculation with cryopreserved cells

A major challenge for adapting our previously established suspension culture protocol [13, 14] to full GMP is the initially required 2D pre-culture, which is poorly controlled, manual handling-dependent and, in particular, incompatible with a closed system approach, which would be highly favorable for the full GMP bioprocessing of hPSCs in a clean room setting [34, 35].

**Fig. 5:**
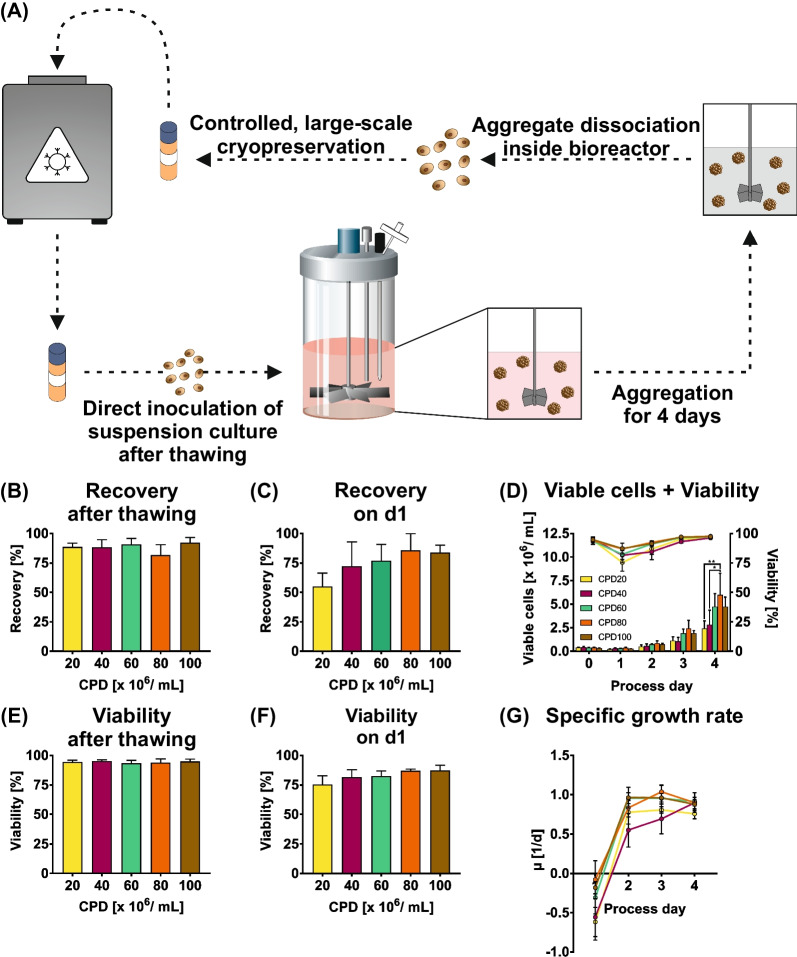
Cryopreservation of suspension-derived hPSCs. **A** Intermediate cryopreservation of suspension culture-derived cells was established to fully enable matrix-free cultivation of hPSCs in stirred tank bioreactors. After 3 passages of each 4 days, cells were harvested after dissociation inside the bioreactor and cryopreserved in different cell densities as single cells. After thawing, these cells were directly transferred to bioreactors for an additional 4 days of cultivation. The cryopreservation cell densities (CPDs) tested were 20 (CPD20, yellow), 40 (CPD40, purple), 60 (CPD60, dark green), 80 (CPD80, dark orange) and 100 × 10^6^ cells/mL (CPD100, brown) (n = 3). **B** The recovery after thawing was calculated as a ratio of the amount of cryopreserved cells and the viable cell count after thawing. **C** Recovery on d1 was calculated as a ratio of the amount of the starting cell number on process day 0 (aimed to be at 0.5 × 10^6^ cells/mL) and the viable cell count on process day 1. **D** Viable cell density (bar chart) and viability (line graph) throughout 4 process days of cultivation after thawing cells cryopreserved at different cell densities. **E** Viability of cells directly after thawing. **F** Viability of cells on process day 1 (corresponding to line graph in D). **G** Specific growth rate µ calculated based on viable cell densities. Results are presented as mean ± SEM

As outlined in the Fig. 5A scheme, cryopreservation of suspension-derived hPSCs may provide an expedient solution to these challenges by ousting the need for 2D monolayer cultivation. Such strategy aims at cryopreserved cell banking for the direct inoculation of 3D suspension culture in STBRs by the thawed hPSC cells.

For this purpose, larger amounts of cells have to be cryopreserved in a comparably small volume; we have therefore tested the cryopreservation cell densities (CPD) of 20, 40, 60, 80 and 100 × 10^6^ cells/mL (termed CPD20–CPD100, respectively) in cryogenic vials. To produce the required amount of cells for these experiments, the cells were expanded in the DASbox Mini Bioreactor System for two passages at 150 mL culture scale followed by process upscaling to the 500 mL scale for an additional third passage in the DASGIP Bioblock, thereby reflecting a typical seed train step. Thereafter, aggregates were dissociated (as described before) in the Bioblock system to obtain a single cell solution. For cryopreservation a relatively simple medium composition consisting of 90% E8 medium and 10% DMSO supplemented with the non-ionic surfactant Pluronic F-68 (0.1%) and RI (10 µM) was used.

Firstly, we wanted to investigate cell loss due to necrosis, which may be caused by ice crystallization or osmotic shock and is directly observable after thawing [36, 37]. The post-thawing recovery was calculated as a ratio of the cell count before and after cryopreservation, revealing a recovery rate of 80 – 100% for all conditions tested (Fig. 5B). Cells that did recover from thawing were showing high cell viability of > 97% tested by conventional trypan blue staining (Fig. 5E). However, the major cause of cell loss after cryopreservation is reportedly apoptosis, more precisely anoikis, occurring in the first 12–24 h post-thawing [38–40]. We therefore calculated recovery on d1 (i.e. 24 h post suspension culture inoculation) that is the ratio of the viable cell density on d1 compared to the inoculation density on d0 (Fig. 5C). This assessment revealed a substantial cell loss of up to 45% at CPD20. Interestingly, the cell loss was substantially reduced to 15–30% for the higher freezing densities; these findings were backed-up by respective viability values ranging at 75–85% on d1 (Fig. 5F). However, subsequent cell sample analysis from day 3 onwards notably revealed > 93% cell viability (Fig. 5D), closely reflecting values observed for the conventional (cryopreservation-independent) hPSC cultivation (data not shown).

It must be noted though, that the differential amount of viable cells recovered on d1 post inoculation, substantially impacted on the final cell yields observed on d4 (Fig. 5D) that is: 2.4 (for CPD20-based inoculation), 2.8 (CPD40), 4.7 (CPD60), 6.0 (CPD80) and 4.7 × 10^6^ cells/mL (CPD100). However, despite this early cell loss-dependent reduction in cell yields on d4, the specific growth rate calculated for the recovered cells was stable and reached a maximum of 0.8–1.0 d^−1^ from d2 onwards (Fig. 5G). This underscores that the cryopreservation step, per se, did not affect the growth kinetics of the viable hPSCs. Moreover, our finding that the cryopreservation at higher cell densities promotes viable cell recovery may facilitate the generation of respective cell banks for direct suspension culture inoculation, without the need for excessive cryopreservation capacities.

### Intermediate high-density cryopreservation of suspension-derived cells fosters long-term matrix-free hPSC bioprocessing

We next combined our newly established protocol for the long-term matrix-free suspension culture with intermediate cryopreservation, to test the hypothesis that matrix-dependent 2D culture is entirely dispensable for hPSC bioprocessing. The cell line hHSC_1285 was cultivated for 3 passages at D4 conditions (equivalent to results shown in Fig. 1E) before aggregates were harvested, dissociated and cells were cryopreserved at 100 × 10^6^ cells/mL, representing a suitable CPD. Subsequently, bioreactors were directly inoculated with these cells after thawing.

**Fig. 6:**
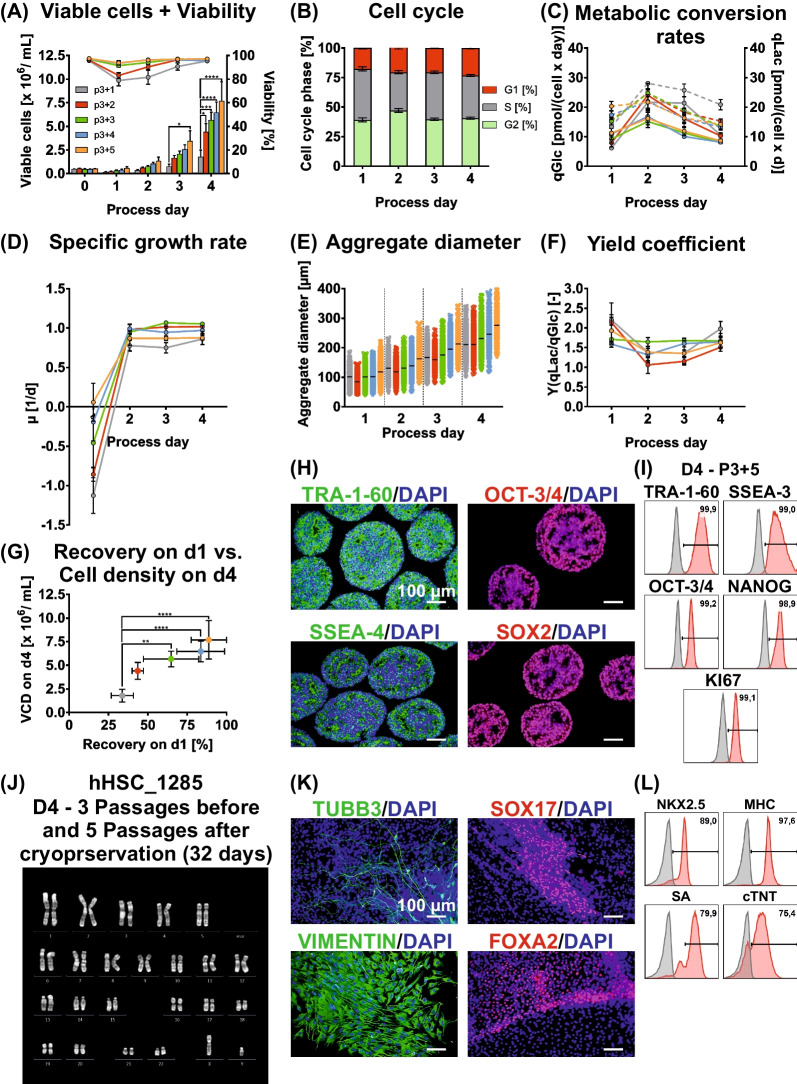
Influence of intermediate cryopreservation of hPSCs on long-term, matrix-free suspension culture in STBRs. **A**–**G** After 3 passages of D4 cultivation (12 days), hHSC_1285 hiPS cells were cryopreserved at 100 × 10^6^ cells/mL for direct inoculation of bioreactors with these cells. Depicted here are the viable cell density (**A**), cell cycle analysis (averaged over 5 passages; **B**), metabolic conversion rates (qGlc as continuous line, qLac as dashed line; **C**), specific growth rate µ (**D**), aggregate diameter distribution (**E**), metabolic yield coefficient (**F**) and the recovery on d1 (**G**) for hHSC_1285 in a D4 cultivation approach over 3 passages (not shown) pre- and 5 passages post-cryopreservation. This resulted in the consecutive passages p3 + 1 (grey), p3 + 2 (red), p3 + 3 (green), p3 + 4 (blue) and p3 + 5 (orange) (n = 3). Results are presented as mean ± SEM. **H** Representative immunocytological stainings of cryosections of hPSC aggregates derived after 3 + 5 passages in D4 (32 days) stained for markers of an undifferentiated state TRA-1-60, OCT-3/4, SSEA-4 and SOX2 (scale bar = 100 µm). (**I**) Exemplary flow cytometry analysis plots for surface markers associated with an undifferentiated state TRA-1-60 and SSEA-3 as well as transcriptions factors OCT-3/4 and NANOG and proliferation marker KI67. Cells were harvested at process endpoint (3 + 5 Passages; 32 days). **J** Karyotype of cells cultivated for 3 + 5 passages in D4 approach (32 days). **K** Undirected differentiation of D4 process-derived aggregates after 5 passages revealed expression of markers representing the three germ layers ectoderm (based on TUBB3), endoderm (based on SOX17 and FOXA2) and mesoderm (based on Vimentin). Scale bar = 100 µm. (**L**) Exemplary flow cytometry analysis plots of cardiomyocyte-specific markers NKX2.5, MHC, SA and cTNT after directed differentiation of cells at the end of passage 3 + 5

Reflecting previous results, the growth kinetic for the first passage after thawing (p3 + 1) was lower compared to the pre-cryopreservation results, yielding an average cell density of 1.8 × 10^6^ cells/mL on d4 (Fig. 6A). Accordingly, the cell viability was at 78% on d1, but stabilized at 95% on d4. After aggregate dissociation and re-inoculation, the cell yield in p3 + 2 was higher compared to the previous passage i.e. 4.4 × 10^6^ cells/mL, but was still lower compared to values before cryopreservation. This was notably associated with a decline in cell viability down to 83% on d1, potentially suggesting a higher sensitivity of the cells to the applied dissociation protocol in consequence to the intermediate cryopreservation step. Nonetheless, cell densities obtained in later passages successively increased to 5.7 (p3 + 3), 6.4 (p3 + 4) and 7.7 × 10^6^ cells/mL (p3 + 5) on d4, respectively, and cell viability remained high and stable at > 95%.

Importantly, the specific growth rate remained high for all passages tested after cryopreservation, reaching the expected maximum between d2 and d4 (Fig. 6D). In p3 + 1, the maximum specific growth rate ranged around ≈0.8 d^−1^, raising to 0.9–1.0 d^−1^ at later passages similar to data observed before cryopreservation. Moreover, cell cycle distribution patterns were also highly consistent with observations before cryopreservation (Fig. 6B; Additional file 1: Figure S10A).

The recovery of cells on d1 (depending on cell survival after re-inoculation) continuously increased from the first to the fifth passage after cryopreservation: 34% (p3 + 1), 44% (p3 + 2), 65% (p3 + 3), 84% (p3 + 4) and 89% (p3 + 5). Notably, this passage-dependent viability on d1 correlated to the cell yield on d4 (Fig. 6G), clearly indicating key importance of early cell survival and aggregation for the subsequent process performance, as previously noted by us [13].

The increase in cell yield from p3 + 1 to p3 + 5 was also reflected by the respective process control parameters i.e. of glucose/lactate (Additional file 1: Figure S10B, C), pH and DO (Additional file 1: Figure S10D), the oxygen concentration in the gas supply (Additional file 1: Figure S10E), and the medium osmolality (Additional file 1: Figure S10F). However, profiles of the cell-specific metabolic conversion rates were matching with those before cryopreservation (Fig. 6C) and the yield coefficient calculated on basis of these conversion rates was ranging from 1.1 to 2.0 for d2–d4 and did not reveal any passage-dependent changes (Fig. 6F).

Finally, we found that the overall aggregate morphology and size distribution patterns were generally retained after cryopreservation (Additional file 1: Figure S10G), but the average aggregate diameter somewhat increased consecutively from ≈210 µm (p3 + 1) to ≈280 µm (p3 + 5) on d4 (Fig. 6E), which similarly observed for GMPDU_8 cells (Additional file 1; Figure S8E).

When adding the 3 passages before and 5 passages after cryopreservation, the hPSCs were cultivated for 32 days in matrix-free suspension culture without indication of pluripotency loss, as demonstrated by immunocytological staining on sections of process-derived hPSC aggregates (Fig. 6H) and by flow cytometry (Fig. 6I). Furthermore, maintenance of the full differentiation potential was validated by undirected differentiation resulting into derivatives representative of all three germ layers (Fig. 6K) and the formation of > 75% cardiomyocytes by our directed differentiation approach (Fig. 6L). Importantly, an unaltered karyotype was revealed (Fig. 6J), together highlighting the great potential of this advanced hPSC-bioprocessing strategy.

## Discussion

In this study, we show that long-term matrix-free 3D suspension culture of hPSCs can entirely replace the need for conventional matrix-dependent 2D culture. The approach allows for the inoculation of cryopreserved, 3D culture-derived hPSCs directly into stirred suspension, which makes matrices required for 2D culture obsolete and enables closed-system manufacturing. This has substantial implications for the development of GMP-compliant large-scale production processes of hPSCs for cell therapies and beyond.

In one aspect, this study underscores that our recently developed high density hPSC bioprocessing strategy [13, 14] is robust, as it builds the basis for the here established multi-passage suspension culture including direct STBR inoculation with cryopreserved cells.

However, our previously established D7 approach revealed that the optimal exponential proliferation of hPSC in aggregates is maintained until day 4 only. The consequent shortening of the cultivation process down to 3 or 4 days (before aggregate dissociation and re-inoculation) proved to be a successful and efficient process modification. As a result, the cumulative ≈77-fold expansion for D3 (in 2 passages; 6 days) and ≈329-fold expansion for D4 (in 2 passages; 8 days) was observed, substantially outperforming the ≈70-fold expansion within 7 days resulting from the D7 protocol. In contrast to the original D7 approach, the optimal exponential growth kinetics of hPSCs was retained for both D3 and D4 over multiple sequential passages.

Assessing the cell population along individual passages, we noted that the constant increase of the resting cell population in G1 phase correlates with the increasing aggregate diameter, even though the average aggregate sizes in D3 or D4 did not exceed the reported critical dimension of 300 µm [30]. However, previous findings in hESC aggregates suggest the formation of local hypoxia in the aggregate core already above 200 µm [41]. Assuming that a similar effects also occurs for the diffusion of nutrients (e.g. glucose) [42], cells within the aggregates may progressively switch to quiescence upon the increase in aggregate diameter along passaging intervals. Such undesirable effects, that may also impact on cells’ subsequent differentiation properties, are apparently minimized by our reduced passage duration followed by subsequent aggregate dissociation and single cell re-inoculation, resulting in a “reset pattern” of the cell cycle.

Notably, a low amount of hPSCs in G1 (and, vice versa, a high amount of S/G2/M phase cells) was suggested as an indicative marker for efficient cardiac differentiation in a 2D monolayer approach [43]. On the other hand, an increased G1 population seems to be associated with genotoxic stress [44], further emphasizing the importance of regular aggregate dissociation for ensuring a stable, high amount of cells in S/G2/M and promoting unrestricted maintenance of hPSCs’ pluripotency over long-term passaging in suspension culture.

Aggregate patterning and morphology for D3 and D4 in this study is closely recapitulating the dimensions observed on days 3–4 of the previously established D7 process. However, after the direct suspension culture inoculation of intermediately cryopreserved cells an increasing mean aggregate diameter was observed along progressive passaging. Such effect may be explained by incomplete aggregate dissociation at passaging, promoting the formation of bigger aggregates directly after re-inoculation, which is then carried over to subsequent passages. This observation may also suggest the occurrence of subtle changes in hPSCs aggregation properties as a consequence of the transition from 2D to 3D cultivation. On the molecular level this may be induced by post-translational modulations of E-cadherin as previously reported [32]. Interestingly, differential expression analysis in this study revealed two cell adhesion-related genes to be upregulated in suspension culture: *CLDN11* and *CDH5*, which encode proteins that are shown to be located at tight/adherens junctions [45, 46] similar to E-Cadherin [47].

Importantly, we show that long-term cultivation of hPSCs in suspension is fully compatible with cells’ optimal proliferation, pluripotency and differentiation properties as well as karyotype stability, revealed by our multiple complementary analyses. However, for further promoting process control and homogeneity upon aggregate dissociation and re-formation, an expedient aggregate dissociation strategy may be required for passaging i.e. by modifying the dissociation time, media and/or mechanical impact and/or by applying appropriate filtration strategies as previously suggested [15–17].

Overall, D3 and D4 share great similarity among each other and with D7, making these modifications in process duration potentially combinable, allowing for more flexible process adaptation to specific project requirements. Respective metabolic patterns, i.e. glucose consumption and lactate production are in check with D7 and with values previously reported for hPSCs [48].

Glucose and lactate concentrations observed in this study remain within the previously suggested, growth-supporting range of > 1.5 mM glucose and < 50 mM lactate [13]. Regarding the cells’ metabolism, we found the upregulation of pathways related to the regulation of insulin secretion in 3D suspension culture-derived samples. This may reflect the uninterrupted stable glucose supply throughout our suspension culture approaches established by perfusion and glucose feeding, in contrast to the 2D cultivation and its batch feeding strategy. Furthermore, an enriched set of DEGs related to *AMPK* signaling was noted in 3D suspension culture, which is shown to be activated by the consumption of ATP [49], potentially promoting glucose uptake and ATP production in response to the energy-consuming, exponential proliferation of the hPSCs under our optimized conditions.

It is finally worth noting that the yield coefficient for glucose/lactate of 1.1–2.0 observed in this study for D3/D4 tallies with published values of 1.4–2.0 [50] and process-related parameters such as medium osmolality, pH and DO almost precisely recapitulated the previously established D7 protocol [13].

Generally, the high similarity between the D3/D4 passaging intervals was corroborated by gene expression analysis. Interestingly, *LEFTY1*/*LEFTY2* and *NODAL* were found to be among the genes differentially upregulated in D4 samples compared to D3, suggesting some impact of the aggregate culture interval on gene expression patterns. It has been suggested that the cell density-dependent accumulation of hPSC-secreted *LEFTY1* may impact on the primitive streak-like patterning at earliest stages of hPSC differentiation [51]. This indicates that the D3 vs. D4 related differences in gene expression, although generally small, may still have relevant consequences for downstream differentiation to be accounted for.

However, we also noted that *LEFTY1* and *LEFTY2* were generally higher expressed in 2D compared to the 3D samples, which may suggest that the expression of these markers is generally sensitive to perturbations of hPSC cultivation.

Gene expression analysis comparing p1 to p5 samples revealed the upregulation of caveolin 1 (*CAV1*) and caveolin 2 (*CAV2*) in p1. This may be related to physical effects such as shear stress, since the role of *CAV1* in response to fluid shear stress has previously been described in endothelial cells [52] and an inhibitory effect of this factor on shear stress-induced anoikis was demonstrated in circulating tumor cells [53]. Metallothioneins, which were previously shown to be responding to fluid shear stress in endothelial cells [54], were similarly found to be upregulated in p1 samples. The downregulation of these fluid shear stress responsive genes in p5 samples may indicate an adaption of hPSCs to the continuous suspension cultivation from earlier to later passage. However, it remains unclear whether this effect is readily completed within the first passages after bioreactor inoculation with 2D adherent culture-derived single cells; investigating such effects will require further analysis of cell samples from earlier passages (p1–p3) to understand the adaptive response of hPSCs to prolonged suspension culture.

The inoculation of STBRs directly with intermediately cryopreserved, suspension-derived hPSCs was straightforward and comparable to the inoculation with 2D-derived cells, obviating the necessity for matrix-dependent monolayer cultivation. Cell viability post-thawing was remarkably high at > 97% in our study compared to previously reported 89.1% [16], while the cell loss on d1 (reflecting apoptosis/anoikis) was piling up to ≈65% in our study, reflecting the ≈70% cell loss recently found at comparable conditions [16]. Importantly, the maximum exponential hPSC growth was swiftly recovered after cryopreservation, rendering the viable cell loss during the first 24 h after inoculation to be the limiting factor for post-cryopreservation process performance.

Besides the Rho-associated kinase (ROCK) inhibitor Y-27632 applied in this study, also the caspase inhibitor Z-VAD-FMK, the p53 inhibitor pifithrin-µ and a small-molecule cocktail proposed by Chen and colleagues have been reported to prevent apoptosis [55–57], but it remains to be investigated whether different inhibitors can further improve the survival of cultured cells after the cryopreservation. A pro-survival conditioning approach has been demonstrated for hESC-derived cardiomyocytes applying heat shock and treatment with IGF-1 before cryopreservation [58], modifications of which may be suitable for undifferentiated hPSCs as well. However, in another aspect, the cell density used for cryopreservation seems not to be a limiting factor in our hands, i.e. tested for up to 100 × 10^6^ cells/mL, whereby hPS cell densities of up to 240 × 10^6^ cells/mL have been applied, but in a microcarrier-based cultivation approach [59].

Maintenance of pluripotency and full differentiation potential is of utmost importance alongside process optimization. Here, the assessment of cells after 8 passages in D4 (32 days) revealed the expression of key markers (i.e. SSEA-3, SSEA-4, TRA-1-60, OCT-3/4, NANOG and KI67) on the protein level both by flow cytometry and immunocytological staining. Moreover, undirected differentiation into the three germ layers and the efficient directed differentiation into cardiomyocytes strongly indicated full retention of the differentiation potential, independent of the D3/D4 passaging interval, the passage number (at least up to passage 8) and the cell lines tested. Furthermore, karyotype stability was maintained at our advanced culture conditions as well.

Our novel protocol substantially exceeds previously reported expansion rates in suspension culture in STBRs. Relevant studies reported values such as 125-fold expansion in 2 passages (14 days) [17] and 925-fold expansion in 3 passages (18 days) [16], which was notably achieved by showing PoC for process upscaling to larger STBR systems. Moreover, a 1100-fold expansion in 3 passages (11 days) [15] was revealed based on cumulative calculations without process scale-up. Applying respective calculations to a hypothetically combined D3 (1 passage; 3 days) and D4 (2 passages; 8 days) approach (total duration of 11 days) a cumulative ≈2880-fold expansion is revealed, highlighting the superior performance of our protocol.

Notably, a recent report demonstrated 93.8-fold expansion of hPSCs in a vertical-wheel bioreactor in a process inoculated with low seeding densities (0.36 × 10^5^ cells/mL vs. 5 × 10^5^ cells/mL in this study) [60]. The application of inoculation cell densities lower than the one applied in this study represents a potential strategy that has previously also been applied for STBR-based cultivation of hPSCs [16, 17], as the inoculation cell density has not been further investigated with the established high density cultivation protocol [13].

Additional calculations by Kwok et al. [17] reported a cumulative cell yield of ≈10^15^ hPSCs over 7 passages (49 days); here we demonstrated a cumulative cell yield of ≈4.7 × 10^15^ hPSCs by 8 sequential passages via the D4 protocol within a substantially shorter time (32 days; with intermediate cryopreservation after the third passage).

By demonstrating the partially automated, bioreactor-based hPSC aggregate dissociation our approach supports progress towards a fully GMP-compliant process. In a recent approach, Huang and colleagues [16] applied bags that were sterile welded to the bioreactor for enabling “closed system-like” connection of cells/aggregate suspensions with respective media/buffer reservoirs, required for the cell dissociation and passaging process, but open-handling steps i.e. for the cells to be transferred into conical tubes for centrifugation-based collection were still required.

Here, for mimicking closed system-compatible handling steps, we have applied a tubing-connected inoculation bottle for cell/reagent transfer from the biosafety cabinet to the bioreactor (Additional file 1: Figure S1). Based on these developments, the future application of fully automated, closed system-compatible devices (e.g. via counterflow centrifugation or alternative strategies) may be implicated for enabling fully closed process conditions in line with GMP and clean room requirements [16, 59].

Previous estimations suggest that culture scales of 1,000 to 2,000 L will be required in the future to accommodate the anticipated demands of 10^11^–10^14^ cells per year per (an entire) therapeutic field [13, 61]. However, the successful upscaling of our D4 approach based on our here presented data, e.g. by stepwise seed train that is from 150 mL to 2 L to 30 L over 3 passages in 12 days, is estimated to result in the production of 2.72 × 10^11^ cells; if successful, this would only require 1.5–3% of the aforementioned culture scale estimations to fuel respective cell demands.

Although most lineage-specific differentiation approaches for hPSCs are still lagging behind the cell yields and culture efficiency demonstrated at the pluripotent state, our current results and these (simplified) calculations are emphasizing the enormous potential of our newly developed protocol for enabling economically viable, “off-the-shelf” production of hPSCs and their respective derivatives.

## Conclusions

In conclusion, this work demonstrates the entire matrix-free suspension-only culture of hPSCs, while setting new efficiency standards for hPSC bioprocessing in STBRs and promoting process upscaling via seed train and closed system bioprocessing, which is a game-changer for therapeutic cell production. Based on the here demonstrated process strategy, the currently applied closed system-like handling steps could be further automated by closed system-compatible devices such as counter-flow centrifuges to enable full compliance with GMP and clean room requirements. The implemented cryopreservation of suspension-derived hPSC could potentially enable the generation of large, off-the-shelf usable cell stocks for promoting the swift clinical production of lineage-specific cells.

### Supplementary Information

**Additional file 1**. Supplementary information.

**Additional file 2**. RNAseq data summary: D3_P5_vs_D3_P1_2factor_m_DESeq2_result_overview.

**Additional file 3**. RNAseq data summary: D4_P1_vs_D3_P1_2factor_m_DESeq2_result_overview.

**Additional file 4**. RNAseq data summary: D4_P5_vs_D3_P5_2factor_m_DESeq2_result_overview.

**Additional file 5**. RNAseq data summary: D4_P5_vs_D4_P1_2factor_m_DESeq2_result_overview.

**Additional file 6**. RNAseq data summary: P5_vs_P1_2factor_m_DESeq2_result_overview.

## Data Availability

The RNAseq data is available in the ScienceDB repository [DOI: https://doi.org/10.57760/sciencedb.16061]. Other datasets generated and/or analysed during this study as well as the code used are available from the corresponding author on reasonable request. This study did not generate new unique reagents.

## Abbreviations

2D: Two-dimensional (adherent cell culture)
3D: Three-dimensional (suspension cell culture)
ANOVA: Analysis of variance
BSA: Bovine serum albumin
CDM3: Chemically defined medium, 3 components
CHIR99021: Selective GSK-3 inhibitor; WNT activator
CPD: Cryopreservation cell density
D3: High density bioprocessing approach for 3 days
D4: High density bioprocessing approach for 4 days
D7: High density bioprocessing approach for 7 days
DAPI: 40,6-Diamidino-2-phenylindole
DEG: Differentially expressed gene
DMSO: Dimethyl sulfoxide
DO: Dissolved oxygen concentration [%]
E8: Essential 8 medium
GMP: Good manufacturing practice
hPSC: Human pluripotent stem cell
IWP2: Inhibitor of WNT production 2
ML: Monolayer (adherent cell culture)
PBS w/o: Phosphate buffered saline without Ca^2+^/Mg^2+^
PBS +: Phosphate buffered saline with Ca^2+^/Mg^2+^
PCA: Principle component analysis
PFA: Paraformaldehyde
qGlc: Cell-specific glucose consumption rate [pmol/(cell × d)]
qLac: Cell-specific lactate production rate [pmol/(cell × d)]
RI: Y-27632; Rho-associated coiled-coil kinase inhibitor
SEM: Standard error of the mean
STBR: Stirred tank bioreactor
TBS: Tris-Buffered Saline
Y: Yield coefficient of lactate from glucose
µ: Specific growth rate [d^−^^1^]
