# Supplementary material for: Matrix-free human pluripotent stem cell manufacturing by seed train approach and intermediate cryopreservation
Source: Stem Cell Res Ther. 2024 Mar 25;15:89. doi: 10.1186/s13287-024-03699-z (PMC10964510; doi:10.1186/s13287-024-03699-z)
Supplement: Supplementary file 1 — Additional file 1. Supplementary information. [file 13287_2024_3699_MOESM1_ESM.pdf]

Supplemental figures:

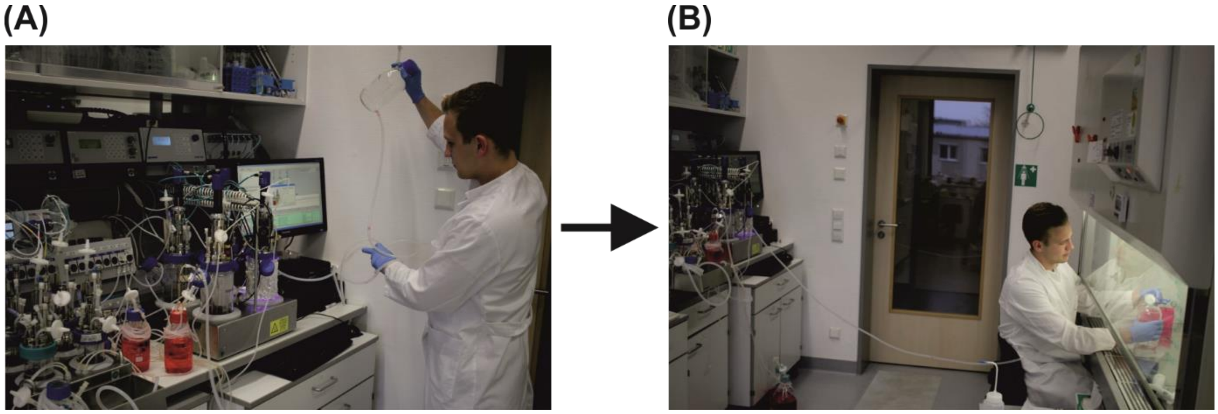

Figure S1: Application of an inoculation bottle for process initiation and cell harvest/dissociation. The inoculation bottle is connected to the bioreactor via a long tubing, allowing the gravity-fed transfer of media/reagents and cells into the bioreactor (A) while on the other hand sterile handling in the biosafety cabinet (B).

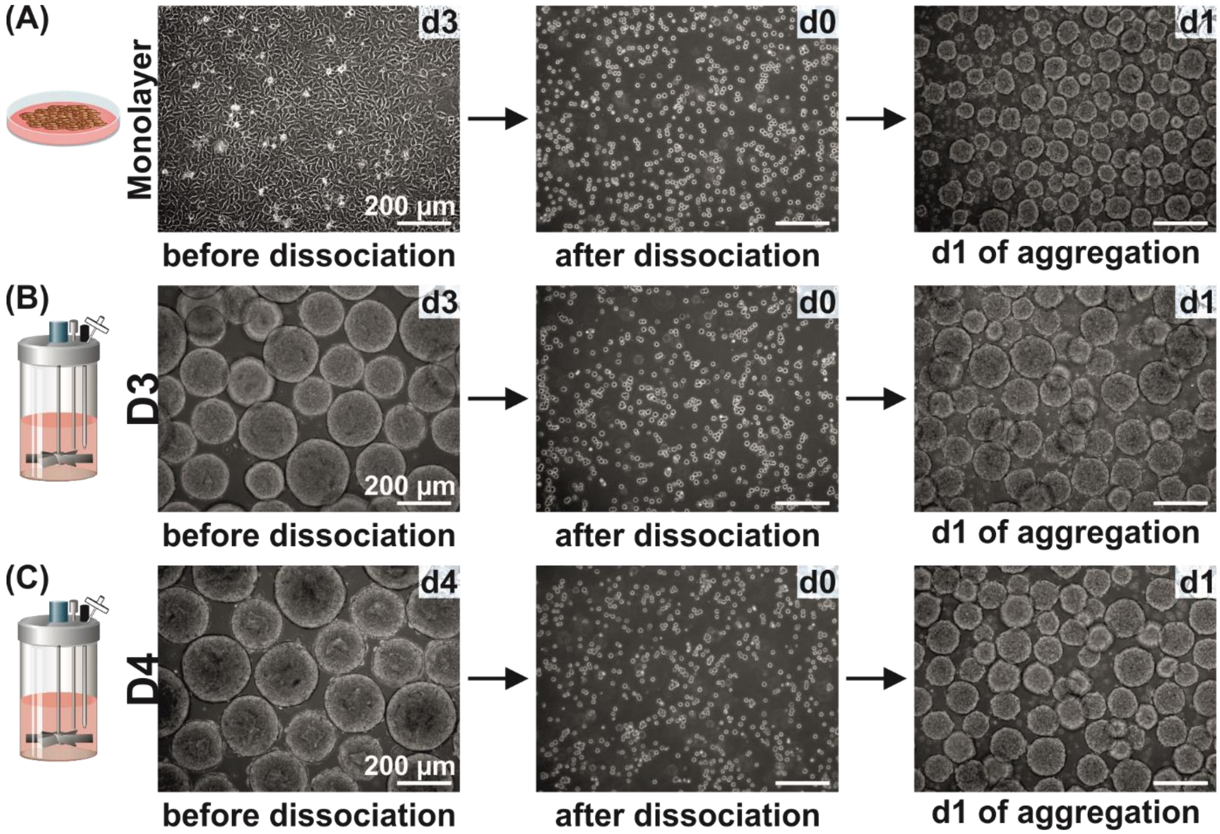

Figure S2: Comparison of different dissociation strategies of human pluripotent stem cell (hPSC) aggregates. Exemplary light microscopic pictures of cells or aggregates before dissociation (left), after dissociation to single cells (middle) and after 1 day of cultivation (right). Detachment of monolayer (ML) cultivated cells on day 3 is shown in A, while dissociation of suspension-derived aggregates after 3 days (D3) and 4 days (D4) are shown in B and C (Scale bar = 200 µm).

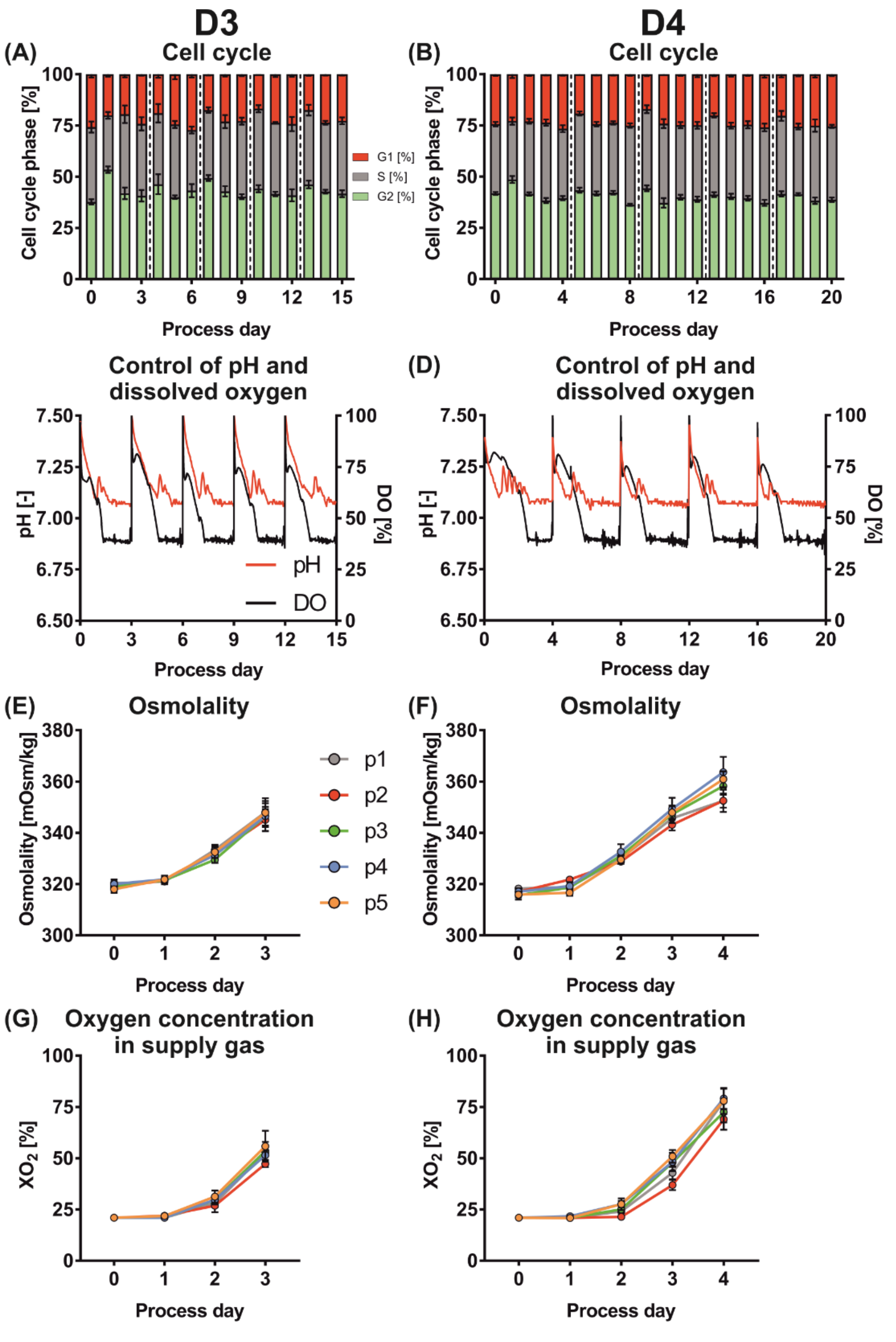

Figure S3: Influence of cultivation interval on cell cycle phase and control of process parameters. A-B: Cell cycle analysis over 5 passages for the 3 day (D3) and the 4 day (D4) cultivation interval. In between dissociation of aggregates is indicated by the vertical, dashed line. C-D: Representative online process parameter measurement of pH (red) and dissolved oxygen (DO) concentration (black) over 5 passages for both the D3 and the D4 approach. E-F: Process-dependent osmolality of the cultivation medium, which gradually increases as a result of base addition for pH control, over 5 passages. G-H: Concentration of oxygen in the gas supply (XO_2) of the bioreactor over 5 passages. XO_2 gradually increases for DO control as cells metabolize more oxygen. Depicted are consecutive passages p1 (grey), p2 (red), p3 (green), p4 (blue) and p5 (orange) (n = 3 – 11). Results are presented as mean ± SEM.

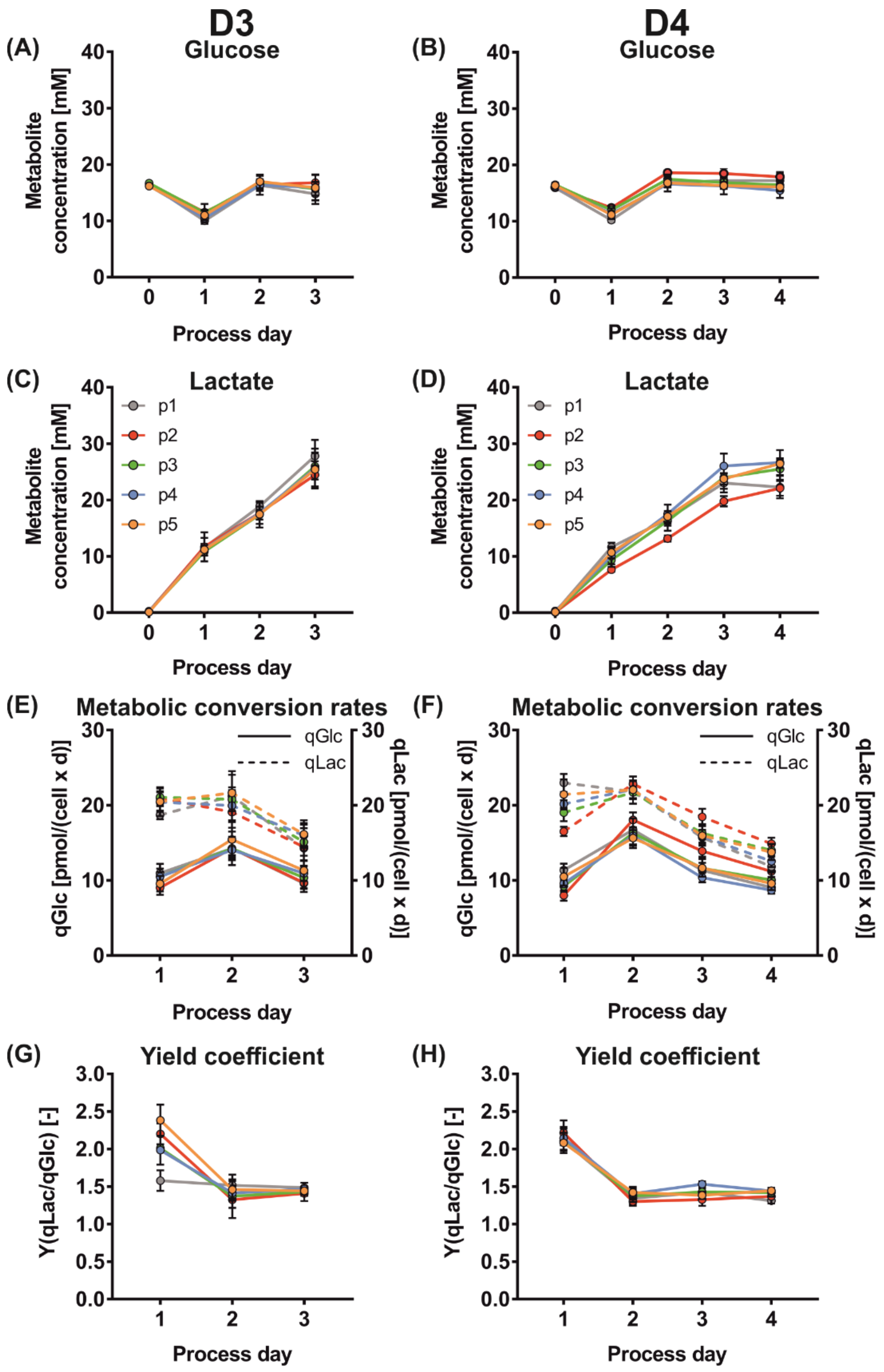

Figure S4: Influence of cultivation interval on hPSCs' metabolic activity. A-D: Process-dependent metabolite profiles of glucose (A-B) and lactate (C-D) for cultivation intervals D3 and D4 in consecutive passages p1 (grey), p2 (red), p3 (green), p4 (blue) and p5 (orange) (n = 3 – 11). E-F: Values for metabolic conversion rates were calculated based on metabolite profiles and viable cell densities and are shown as continuous line for the cell-specific glucose consumption rate (qGlc) and as dashed line for the cell-specific lactate production rate (qLac). G-H: The metabolic yield coefficient was calculated as a ratio of qLac and qGlc. Results are presented as mean ± SEM.

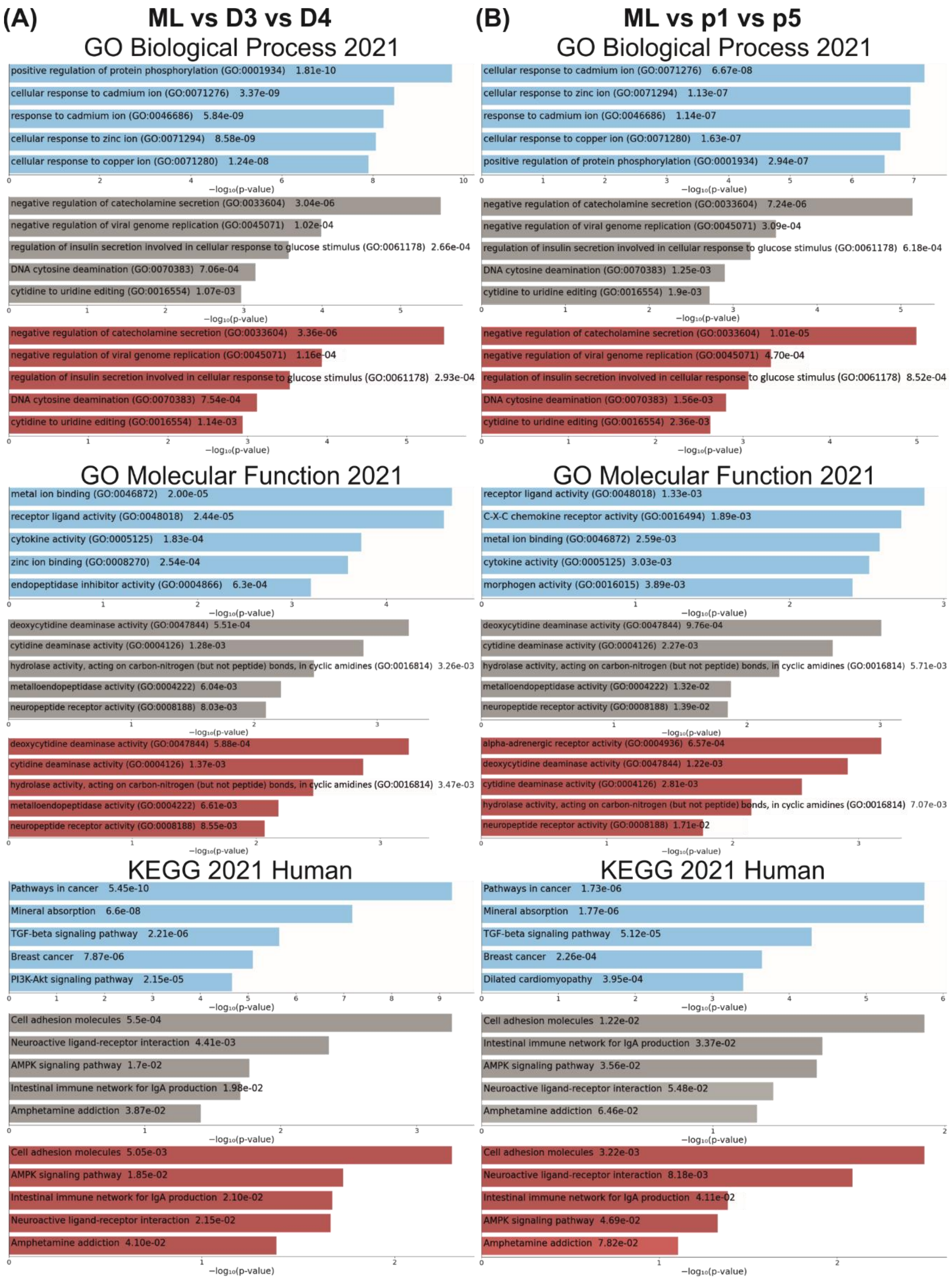

Figure S5: Gene set enrichment analysis of differentially expressed genes (DEGs) comparing adherent and suspension culture-derived samples. Gene set enrichment analysis via Enrichr for biological processes, molecular functions and pathways using the Gene Ontology (GO) and the Kyoto Encyclopedia of Genes and Genomes (KEGG) for DEGs in: ML (blue), D3 (grey) and D4 (red)-derived samples (A); ML (blue), p1 (grey) and p5 (red)-derived samples (B).

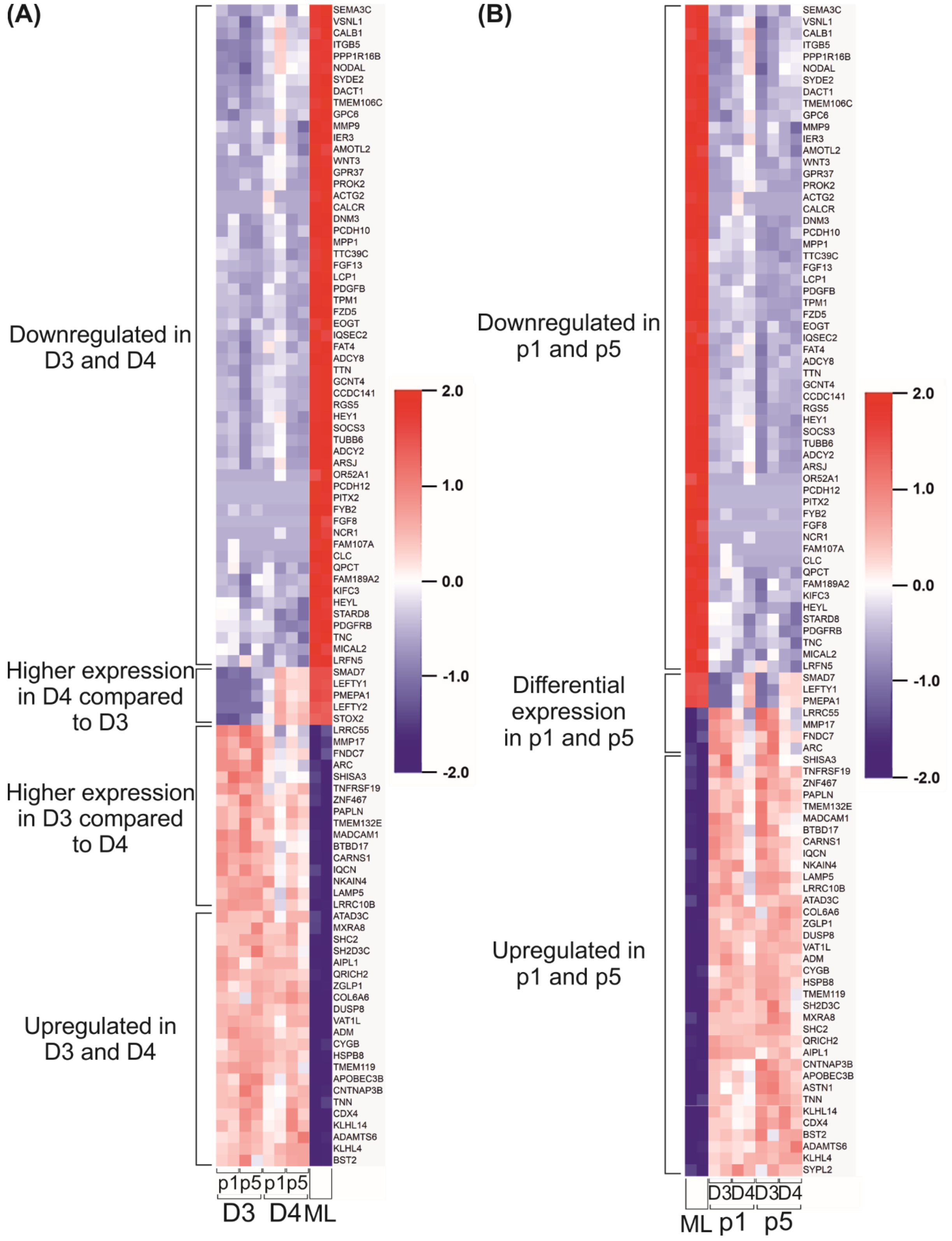

Figure S6: Clustering and heat map analysis of DEGs in comparison of adherent and suspension culture-derived samples. A: Clustering and heat map analysis of the 100 most significant ($p < .00024$) DEGs observed in comparison of ML, D3 and D4 samples. B: Clustering and heat map analysis of the 100 most significant ($p < .00025$) DEGs in comparison of ML, p1 and p5 samples.

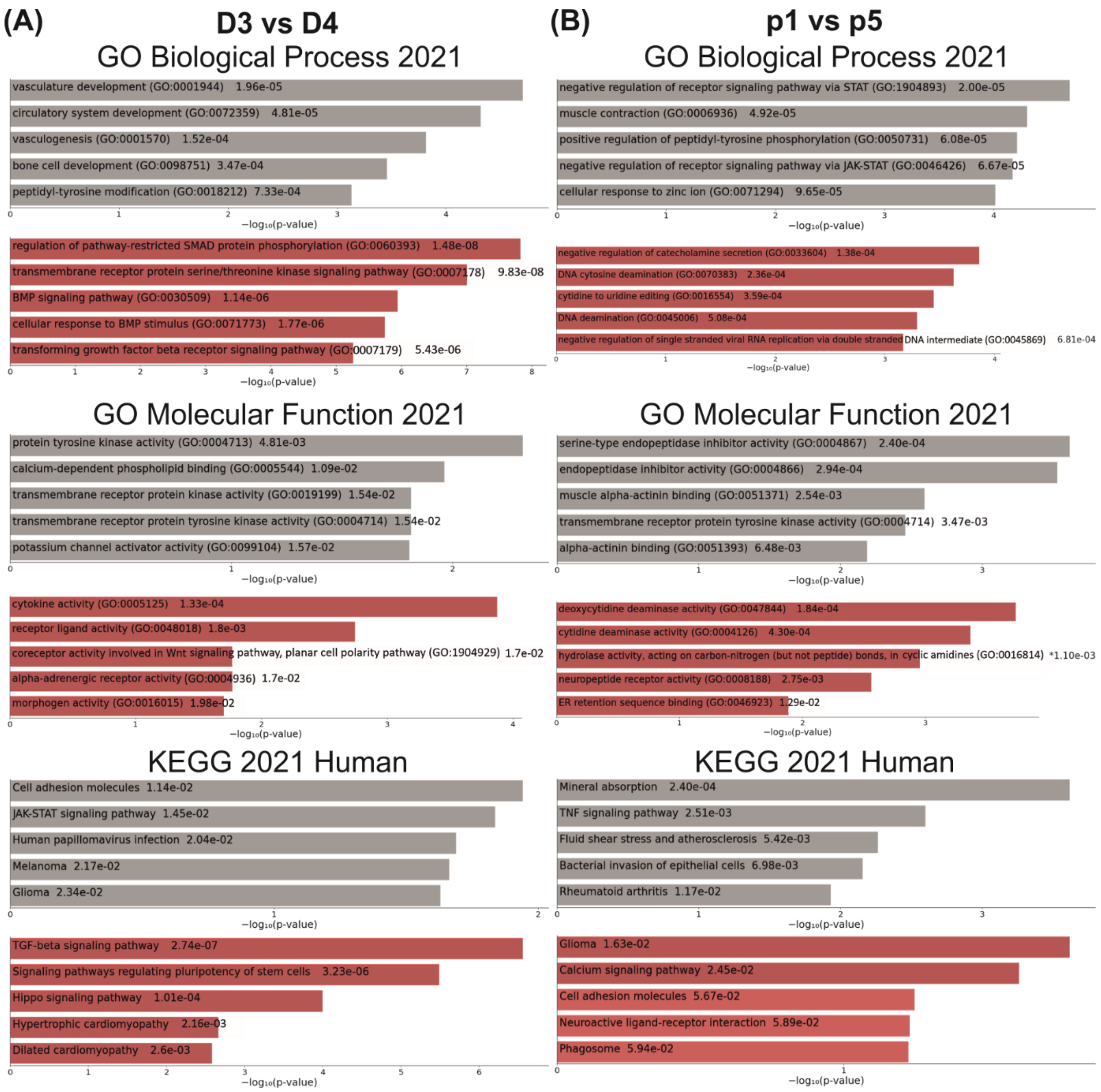

Figure S7: Gene enrichment analysis of DEGs in comparison of adherent and suspension culture-derived samples. A: Gene set enrichment analysis via Enrichr for biological processes, molecular functions and pathways using the GO and KEGG resources for DEGs found for D3 (grey) and D4 (red)-derived samples. B: Gene set enrichment analysis via Enrichr for biological processes, molecular functions and pathways using the GO and KEGG resources for p1 (grey) and p5 (red) derived samples.

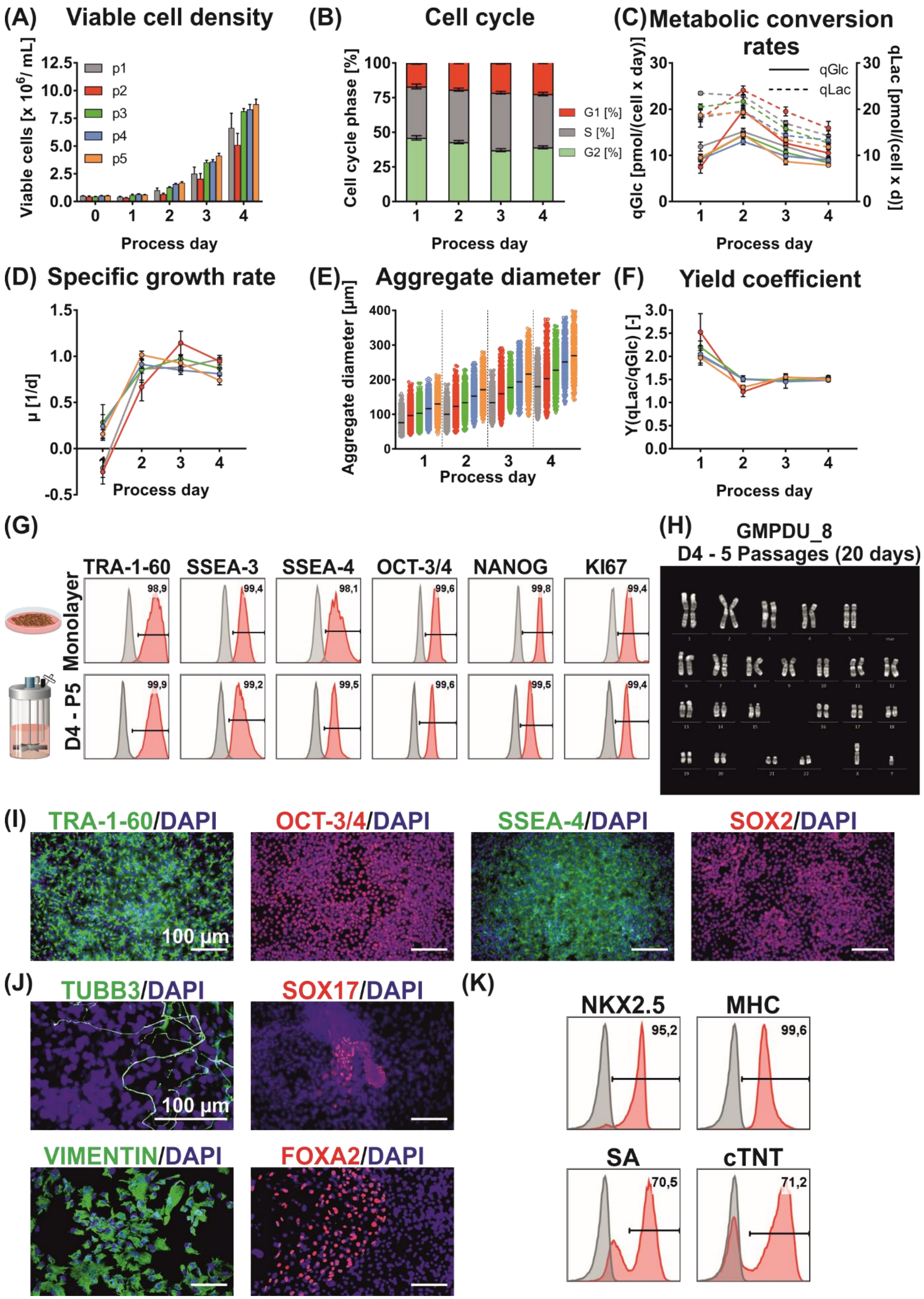
(A) Viable cell density
Viable cells [x 10^6/ mL]
p1
p2
p3
p4
p5
Process day
(B) Cell cycle
Cell cycle phase [%]
G1 [%]
S [%]
G2 [%]
Process day
(C) Metabolic conversion rates
qGlc [pmol/(cell x day)]
qLac [pmol/(cell x d)]
qGlc
qLac
Process day
(D) Specific growth rate
µ [1/d]
Process day
(E) Aggregate diameter
Aggregate diameter [µm]
Process day
(F) Yield coefficient
Y(qLac/qGlc) [-]
Process day
(G)
TRA-1-60
SSEA-3
SSEA-4
OCT-3/4
NANOG
KI67
Monolayer
D4 - P5
98,9
99,4
98,1
99,6
99,8
99,4
99,9
99,2
99,5
99,6
99,5
99,4
(H) GMPDU_8
D4 - 5 Passages (20 days)
(I)
TRA-1-60/DAPI
OCT-3/4/DAPI
SSEA-4/DAPI
SOX2/DAPI
100 µm
(J)
TUBB3/DAPI
SOX17/DAPI
100 µm
VIMENTIN/DAPI
FOXA2/DAPI
(K)
NKX2.5
MHC
95,2
99,6
SA
cTNT
70,5
71,2

Figure S8: Confirmation of the D4 cultivation protocol with the independent hiPS cell line GMPDU_8. A-F: Viable cell density (A), cell cycle analysis (5 passages average; B), metabolic conversion rates (qGlc as continuous line, qLac as dashed line; C), specific growth rate µ (D), aggregate diameter distribution (E) and the metabolic yield coefficient (F) for GMPDU_8 in a D4 cultivation approach over 5 passages. This resulted in the consecutive passages p1 (grey), p2 (red), p3 (green), p4 (blue) and p5 (orange) (n = 3). Results are presented as mean ± SEM. G: Exemplary flow cytometry analysis plots for surface markers associated with an undifferentiated state TRA-1-60, SSEA-3 and SSEA-4 as well as transcriptions factors OCT-3/4 and NANOG and proliferation marker KI67. Cells harvested at process endpoint (5 Passages; 20 days) were compared to monolayer-derived cells used for inoculation of the processes. H: Karyotype of cells cultivated for 5 passages in D4 approach (20 days). I: Representative immunofluorescence pictures of aggregate-derived, single cell-dissociated hPSCs re-seeded after 5 passages in D4 (20 days) and stained for markers TRA-1-60, OCT-3/4, SSEA-4 and SOX2 (scale bar = 100 µm). J: Undirected differentiation of D4 process-derived aggregates after 5 passages revealed the expression of markers representing the three germ layers ectoderm (based on TUBB3), endoderm (based on SOX17 and FOXA2) and mesoderm (based on Vimentin). Scale bar = 100 µm. K: Exemplary flow cytometry analysis plots of cardiomyocyte-specific markers NKX2.5, MHC, SA and cTNT after directed differentiation of D4-derived cells at the end of passage 5.

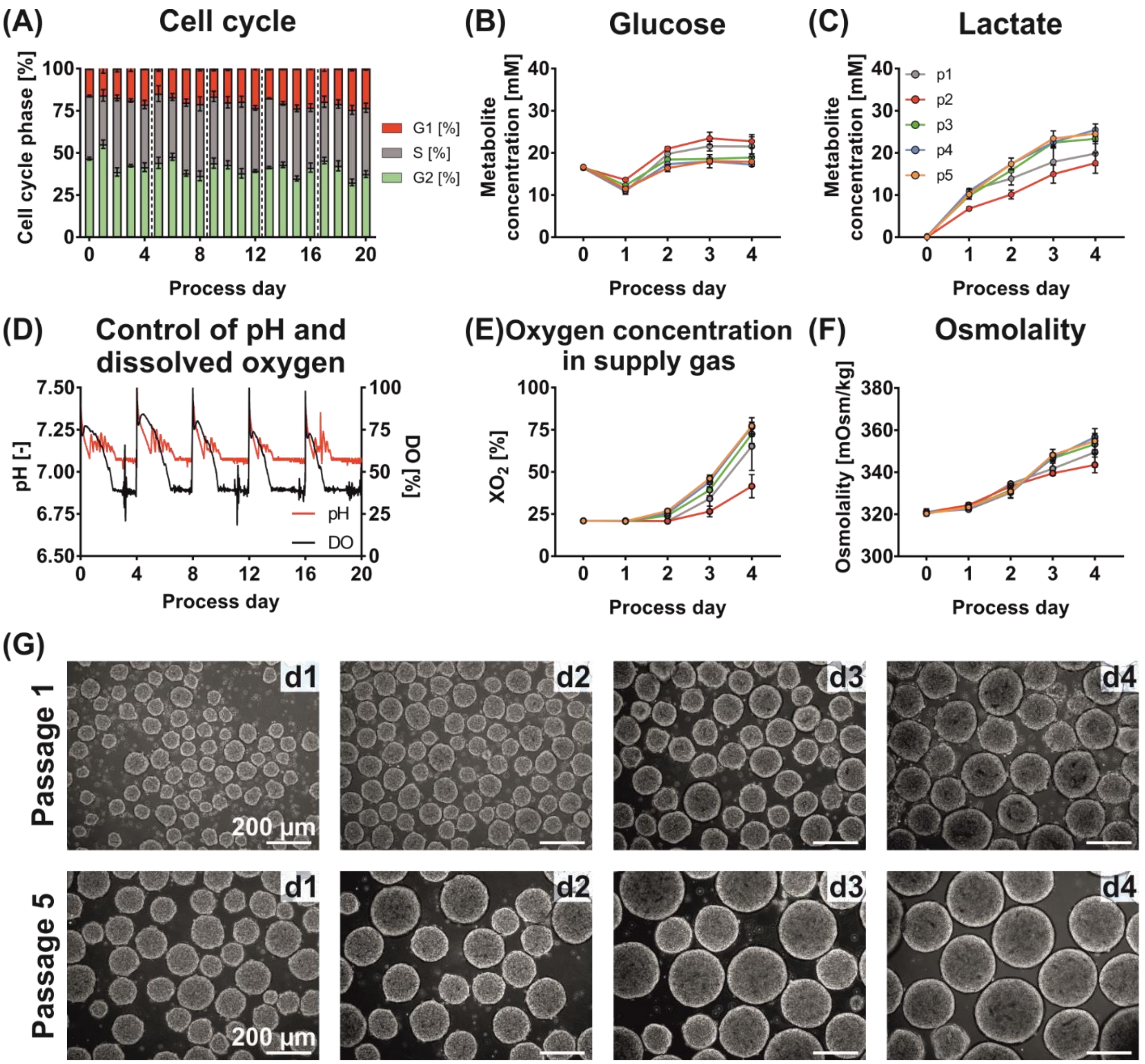

Figure S9: Influence of matrix-free, long-term suspension culture on process performance of GMPDU_8. A: Cell cycle analysis over 5 passages. In between dissociation of aggregates is indicated by the vertical, dashed line. B-C: Process-dependent metabolite profiles of glucose (B) and lactate (C). D-F: Representative online process parameter measurement (D) of pH (red) and DO (black), process-dependent osmolality of the cultivation medium (E) and the concentration of oxygen in the gas supply of the bioreactor XO_2 over 5 passages (F). Depicted are consecutive passages p1 (grey), p2 (red), p3 (green), p4 (blue) and p5 (orange) (n = 3). Results are presented as mean ± SEM. G: Exemplary light microscopic pictures of process derived aggregate samples on all process days of passage 1 and passage 5 (scale bar = 200 µm).

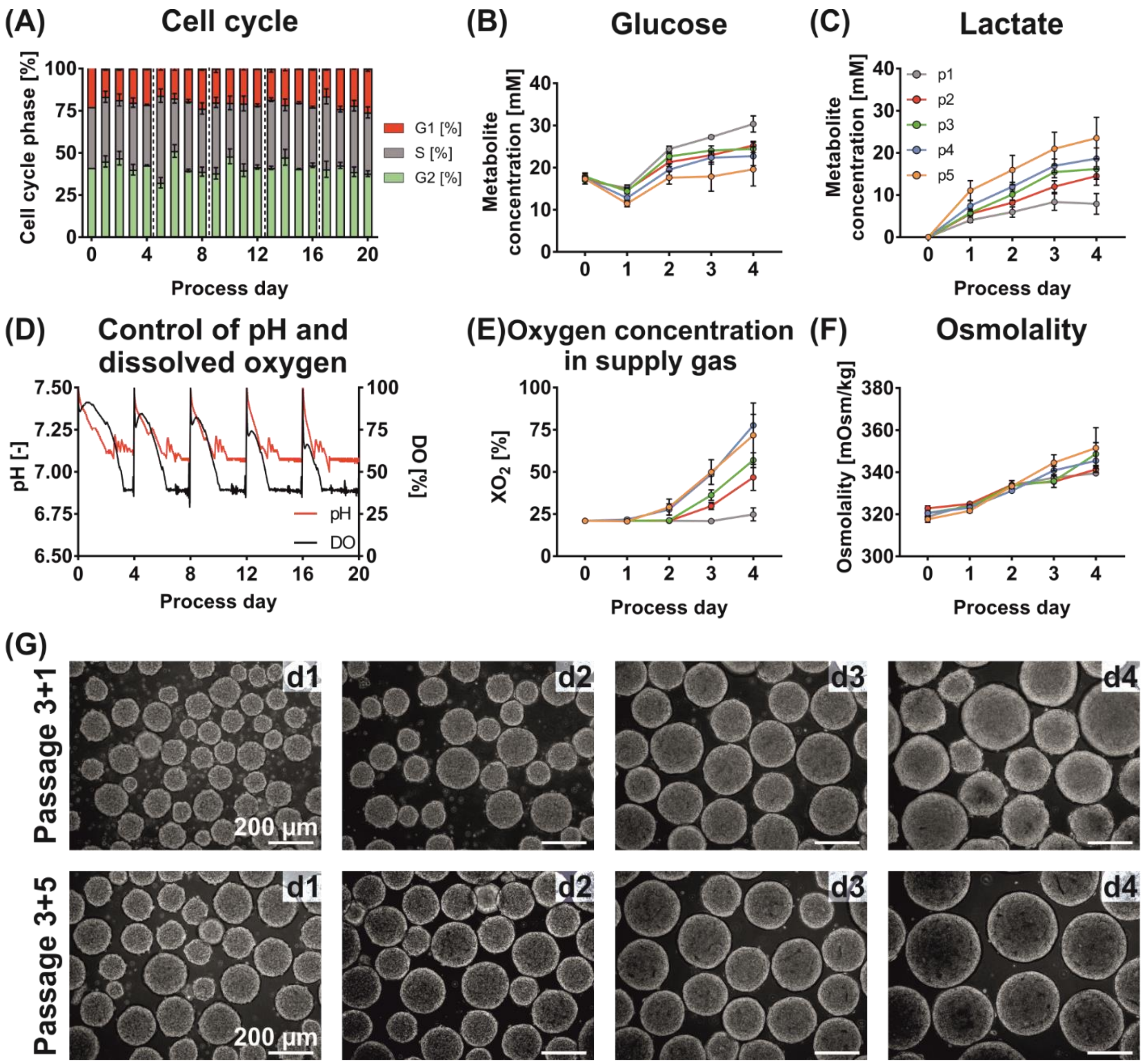

Figure S10: Long-term, matrix-free process performance of hHSC_1285 after intermediate cryopreservation. A: Cell cycle analysis over 5 passages after cryopreservation. In between dissociation of aggregates is indicated by the vertical, dashed line. B-C: Process-dependent metabolite profiles of glucose (B) and lactate (C). D-F: Representative online process parameter measurement (D) of pH (red) and DO (black), process-dependent osmolality of the cultivation medium (E) and the concentration of oxygen in the gas supply of the bioreactor XO_2 (F) over 5 passages after cryopreservation. Depicted are consecutive passages p3+1 (grey), p3+2 (red), p3+3 (green), p3+4 (blue) and p3+5 (orange) (n = 3). Results are presented as mean ± SEM. G: Exemplary light microscopic pictures of process derived aggregate samples on all process days of passage 3+1 and passage 3+5 (scale bar = 200 µm).

Supplemental Tables:

Table S1: Composition of E8 medium used in this study

Reagent	Supplier	Final concentration
**E8 used for adherent cell culture**		
DMEM/F-12, HEPES	Thermo Fisher Scientific	N/A (basis medium)
Animal-Free Recombinant Human FGF-basic	Peprotech	100 µg/L
$NaHCO_3$	Merck	0.543 g/L
Na_2SeO_3	Merck	14 µg/L
Insulin solution human	Merck	20 mg/L
Transferrin, human recombinant	Merck	10.7 mg/L
Ascorbic acid 2-phosphate	Merck	64 mg/L
Recombinant Human TGF-β1	Peprotech	2 µg/L
**Additional Supplementation for single cell (re-)inoculation (in 2D adherent and 3D suspension culture)**		
Y-27632 dihydrochloride	Tocris Bioscience	10 µM
**Additional Supplementation for 3D suspension culture**		
Pluronic F-68 Non-ionic Surfactant (10%)	Thermo Fisher Scientific	0.1%
**Additional Supplementation for 3D suspension culture perfusion medium**		
D-(+)-Glucose Hybri-Max	Merck	3 g/L
L-Glutamine (200 mM)	Thermo Fisher Scientific	2 mM

Table S2: Perfusion rates at respective process days

Process day	Perfusion rate [1/d]
0-1	0
1-2	1
2-3	1.5
3-4	3

Table S3: Directly labeled antibodies used for flow cytometry analysis of markers associated with an undifferentiated state

Name	Class	Species recognition	Conjugate	Supplier	Dilution
Anti-SSEA-3	IgM	Human	DyLight 650	Thermo Fisher	1:250
Anti-SSEA-4	IgG1	Human	VioBlue	Mitlenyi Biotec	1:25
Anti-TRA-1-60	IgG1	Human	PE	Mitlenyi Biotec	1:25
Anti-OCT-3/4 Isoform A	IgG1	Human/mouse	PE	Mitlenyi Biotec	1:25
Anti-NANOG	IgG1	Human	APC	Mitlenyi Biotec	1:25
Anti-KI67	IgG1	Human/mouse	PerCP-Vio700	Mitlenyi Biotec	1:25

Table S4: Controlled-rate freezer protocol

Step	Description
1	Wait at 4 °C
2	Load freezing tubes into controlled-rate freezer
3	-1.2 °C/min to -4 °C
4	Hold at -4 °C for 10 minutes
5	-25 °C/min to -40 °C
6	10 °C/min to -12 °C
7	-1 °C/min to -40 °C
8	-10 °C/min to -90 °C

Continued on next page

Continued

Step	Description
9	Wait at -90 °C
10	Transfer freezing tubes to -150 °C for long-term storage

Table S5: Composition of CDM3 for directed cardiomyogenic differentiation

Reagent	Supplier	Final concentration
Human Serum Albumin	ScienCell	495 mg/L
L-Ascorbic acid 2-phosphate	Merck	213 mg/L
RPMI 1640	Thermo Fisher Scientific	N/A (basis medium)
Medium supplementation for d0 – d1 of differentiation		
CHIR99021	Institute for Organic Chemistry, Leibniz University Hannover	5 µM
Medium supplementation for d1 – d3 of differentiation		
IWP2	Tocris Bioscience	5 µM

Table S6: Primary and secondary antibodies used for flow cytometry analysis of cardiomyocyte-specific markers

Name	Class	Species	Clonality	Supplier	Dilution
**Primary Antibodies**					
Anti-Cardiac Troponin T	IgG1	Mouse	Monoclonal	Thermo Fisher Scientific	1:200
Anti-Myosin (Sceletal)	IgG1	Mouse	Monoclonal	Merck	1:20
Anti-α-Actinin (Sarcomeric)	IgG1	Mouse	Monoclonal	Merck	1:800
Anti-NKX2.5	IgG	Rabbit	Monoclonal	Cell Signaling	1:800
Mouse IgG1 Isotype	IgG1	Mouse	Monoclonal	Agilent Technologies	1:100
Rabbit IgG Isoytpe	IgG	Rabbit	Polyclonal	Abcam	1:5.000
**Secondary Antibodies**					
Alexa Fluor 647-conjugated Anti-Rabbit IgG (H+L)	F(ab')$_2$ Fragment	Donkey	Polyclonal	Jackson ImmunoResearch	1:200
Cy5-conjugated Anti-Mouse IgG (H+L)	IgG	Donkey	Polyclonal	Jackson ImmunoResearch	1:200
Alexa Fluor 647-conjugated Anti-Mouse IgG (H+L)	IgG	Donkey	Polyclonal	Jackson ImmunoResearch	1:200

Table S7: Composition of undirected differentiation medium per 50 mL

Reagent	Supplier	Amount [mL]
IMDM (Iscove's Modified Dulbecco's Medium)	Thermo Fisher Scientific	40
HyClone Characterized Fetal Bovine Serum (FBS)	Cytiva	10
L-Glutamine (200 mM)	Thermo Fisher Scientific	0.25
MEM Non-Essential Amino Acids Solution (100x)	Thermo Fisher Scientific	0.5
2-Mercaptoethanol	Thermo Fisher Scientific	0.1

Table S8: Composition of TBS, pH 7.6

Reagent	Supplier	Final concentration
Tris(hydroxymethyl)aminomethane	Serva	6,057 g/L
NaCl	Merck	8,607 g/L
dH_2O (Ampuwa)	Fresenius Kabi	N/A (basis medium)

Table S9: Primary and secondary antibodies used for immunocytological staining of pluripotent and undirected differentiated cells

Name	Class	Species	Clonality	Supplier	Dilution
**Primary Antibodies**					
Anti-FOXA2	IgG	Rabbit	Polyclonal	Merck	1:200
Anti-OCT-3/4	IgG2b	Mouse	Monoclonal	Santa Cruz Biotechnology	1:100
Anti-SOX17	IgG	Goat	Polyclonal	R&D Systems	1:200
Anti-SOX2	IgG	Rabbit	Monoclonal	Cell Signaling	1:200
Anti-SSEA-4	IgG3	Mouse	Monoclonal	DSHB	1:100
Anti-TRA-1-60	IgM	Mouse	Monoclonal	Abcam	1:100
Anti-TUBB3	IgG2a	Mouse	Monoclonal	Merck	1:400
Anti-Vimentin	IgG	Rabbit	Monoclonal	Abcam	1:500
Goat IgG Isotype	IgG	Goat	Polyclonal	R&D Systems	1:100
Mouse IgG2a Isotype	IgG2a	Mouse	Monoclonal	Agilent Technologies	1:100
Mouse IgG2b Isotype	IgG2b	Mouse	Monoclonal	Agilent Technologies	1:50
Mouse IgG3 Isotype	IgG3	Mouse	Monoclonal	R&D Systems	1:125
Mouse IgM Isotype	IgM	Mouse	Monoclonal	Agilent Technologies	1:25
Rabbit IgG Isotype	IgG	Rabbit	Polyclonal	Abcam	1:5.000
**Secondary Antibodies**					
Alexa Fluor 488-conjugated Anti-Rabbit IgG (H+L)	IgG	Donkey	Polyclonal	Jackson ImmunoResearch	1:200
Cy3-conjugated Anti-Goat IgG (H+L)	IgG	Donkey	Polyclonal	Jackson ImmunoResearch	1:200
Cy3-conjugated Anti-Mouse IgG (H+L)	IgG	Donkey	Polyclonal	Jackson ImmunoResearch	1:200
Cy3-conjugated Anti-Mouse IgM, µ chain	IgG	Donkey	Polyclonal	Jackson ImmunoResearch	1:200

Table S10: Primary and secondary antibodies used for immunocytological staining of cryosections of hPSC aggregates

Name	Class	Species	Clonality	Supplier	Dilution
**Primary Antibodies**					
Anti-OCT-3/4	IgG2b	Mouse	Monoclonal	Santa Cruz Biotechnology	1:100
Anti-SSEA-4	IgG3	Mouse	Monoclonal	DSHB	1:100
Anti-TRA-1-60	IgM	Mouse	Monoclonal	Abcam	1:100
Anti-SOX2	IgG	Rabbit	Monoclonal	Cell Signaling	1:200
Mouse IgM Isotype	IgM	Mouse	Monoclonal	Agilent Technologies	1:25
Mouse IgG2b Isotype	IgG2b	Mouse	Monoclonal	Agilent Technologies	1:50
Mouse IgG3 Isotype	IgG3	Mouse	Monoclonal	R&D Systems	1:125
Rabbit IgG Isotype	IgG	Rabbit	Polyclonal	Abcam	1:5.000
**Secondary Antibodies**					
Alexa Fluor 488-conjugated Anti-Rabbit IgG (H+L)	IgG	Donkey	Polyclonal	Jackson ImmunoResearch	1:200

Continued on next page

Continued

Name	Class	Species	Clonality	Supplier	Dilution
**Secondary Antibodies**					
Cy3-conjugated Anti-Mouse IgG (H+L)	IgG	Donkey	Polyclonal	Jackson ImmunoResearch	1:200
Cy3-conjugated Anti-Mouse IgM, μ chain	IgG	Donkey	Polyclonal	Jackson ImmunoResearch	1:200

Table S11: Pathways corresponding to DEGs detected via bulk RNA sequencing

**ML vs. 3D**	
**Pathways upregulated in ML**	**Pathways upregulated in 3D**
- *TGF-β* signaling (*LEFTY1*, *ID1*, *PITX2*, *THBS1*, *LEFTY2*, *BMP7*, *NODAL*, *SMAD7*)<br>- *PI3K/AKT* signaling (*PDGFRB*, *CDKN1A*, *ITGB5*, *ANGPT1*, *TNC*, *PDGFB*, *THBS1*, *FGF4*, *TCL1B*, *FGF8*, *LPAR6*, *COL4A3*, *TEK*)<br>- Metallothioneins (*MT2A*, *MT1F*, *MT1G*, *MT1X*, *MT1H*, *MMP9*, *MT1E*)	- Cell adhesion (*CLDN11*, *NTNG2*, *MADCAM1*, *HLA-DQB1*)<br>- *AMPK* signaling (*FBP1*, *CFTR*, *CREB5*)<br>- Insulin secretion (*ADRA2A*, *CFTR*, *FOXA2*)
**D3 vs. D4**	
**Pathways upregulated in D3**	**Pathways upregulated in D4**
- Cell adhesion (*CDH5*, *NTNG2*, *MADCAM1*)<br>- *JAK/STAT* signaling (*PDGFRB*, *CDKN1A*, *THPO*)<br>- Kinase activity (*PDGFRB*, *PKDCC*, *TEK*)	- *TGF-β* signaling and SMAD protein phosphorylation (*LEFTY1*, *LEFTY2*, *BMP7*, *NODAL*, *SMAD7*, *ID1*, *WNT3*)
**p1 vs. p5**	
**Pathways upregulated in p1**	**Pathways upregulated in p5**
- negative regulation of receptor signaling via *JAK/STAT* (*SOCS3*, *CAV1*, *MT1X*)<br>- Metallothioneins (*MT2A*, *MT1F*, *MT1X*)<br>- Fluid shear stress (*CAV1*, *CAV2*, *PDGFB*, *CCL2*)	- DNA deamination (*APOBEC3H*, *APOBEC3B*)
